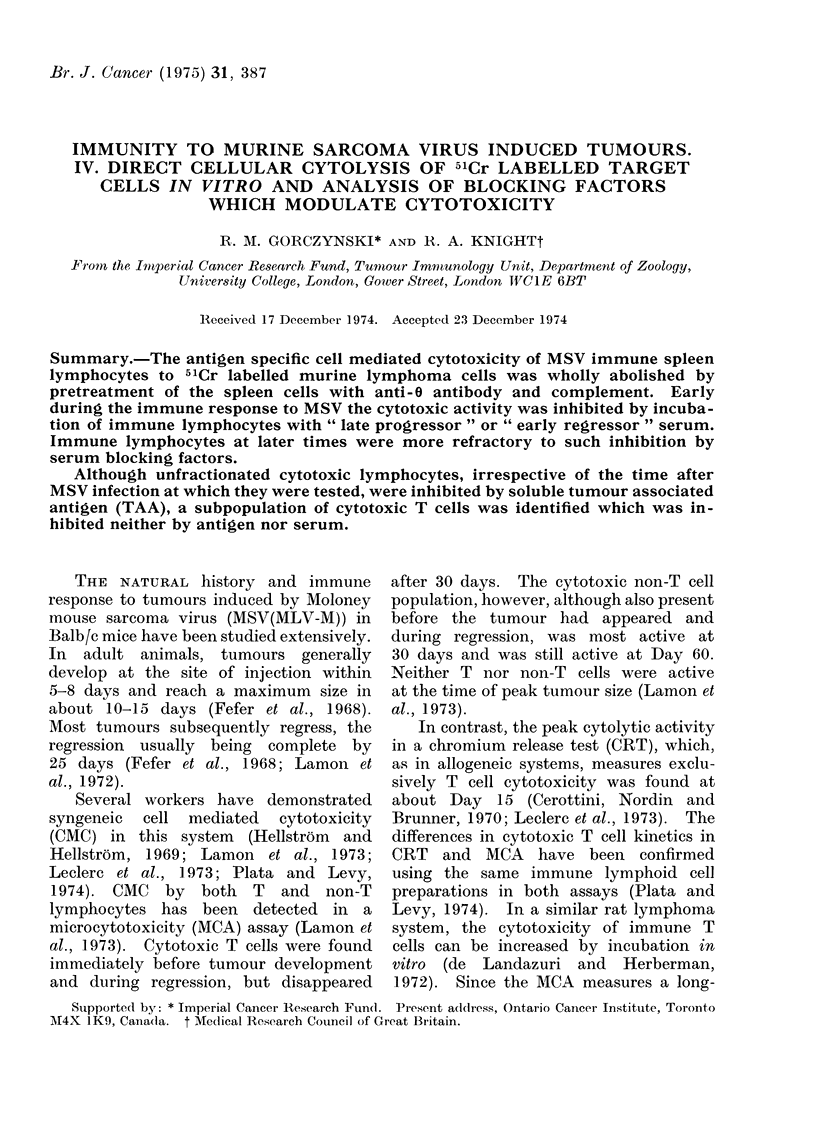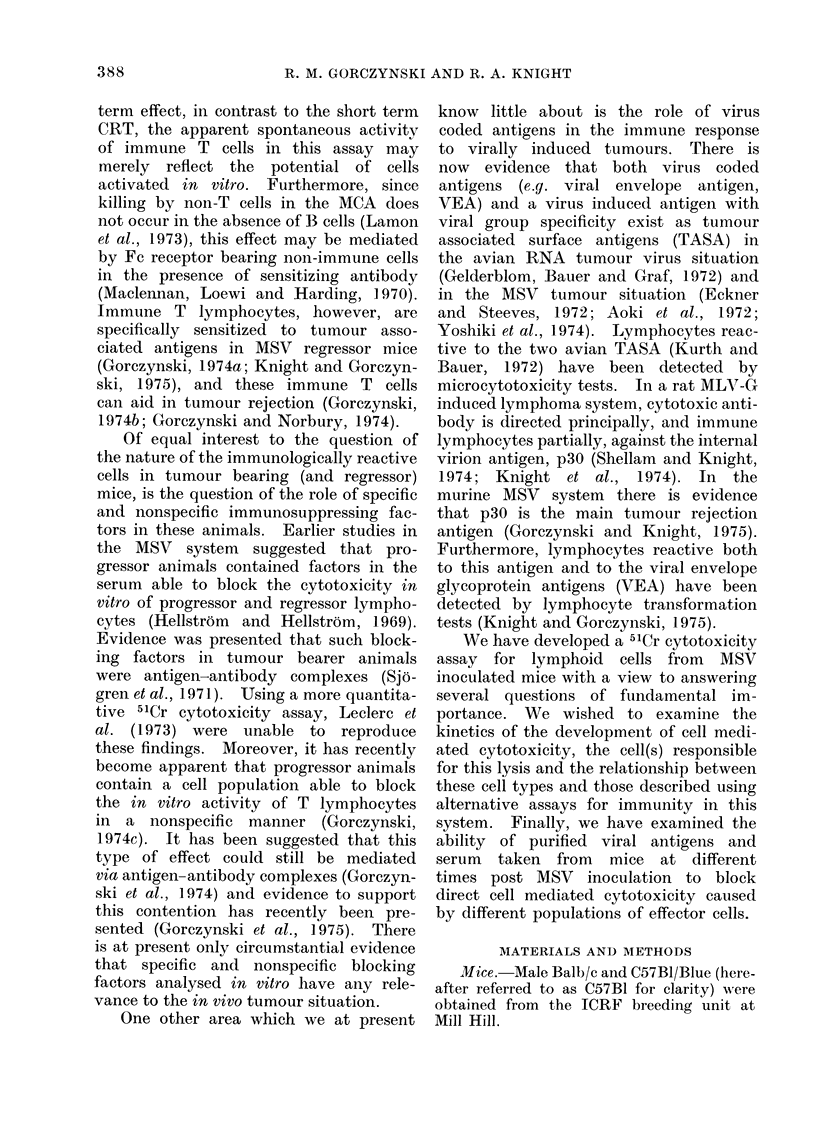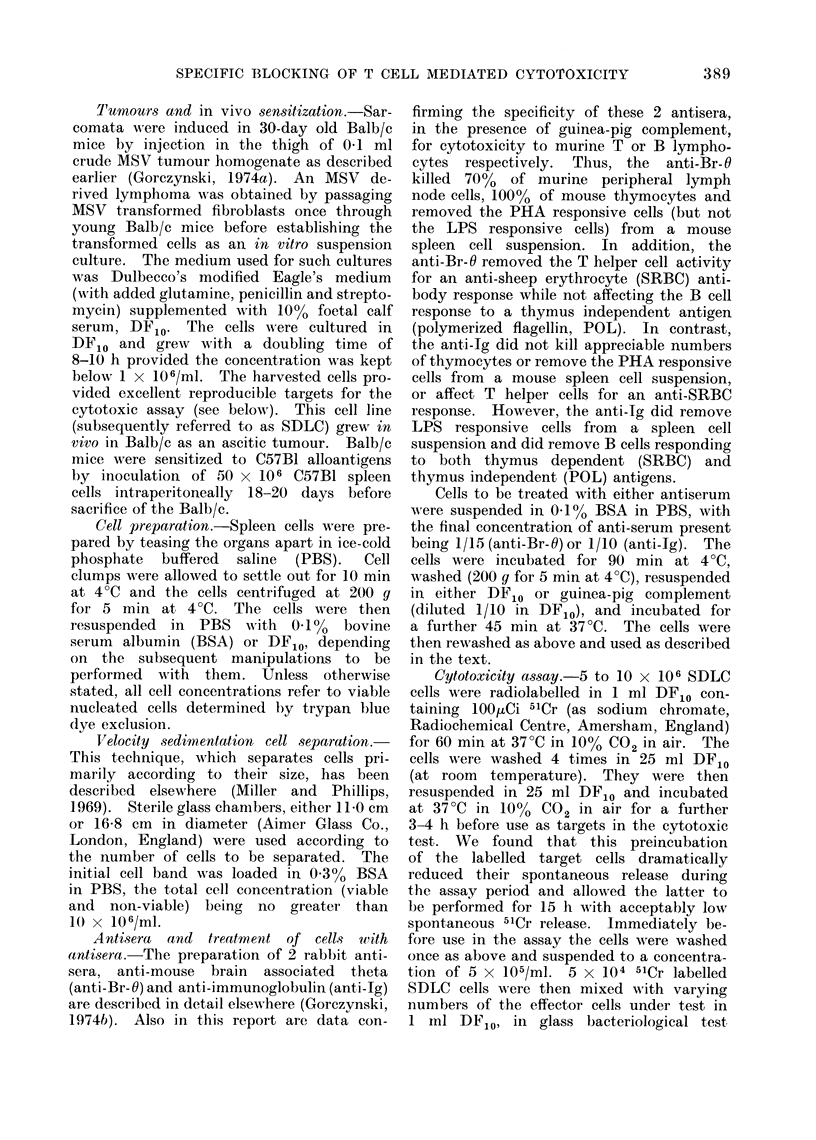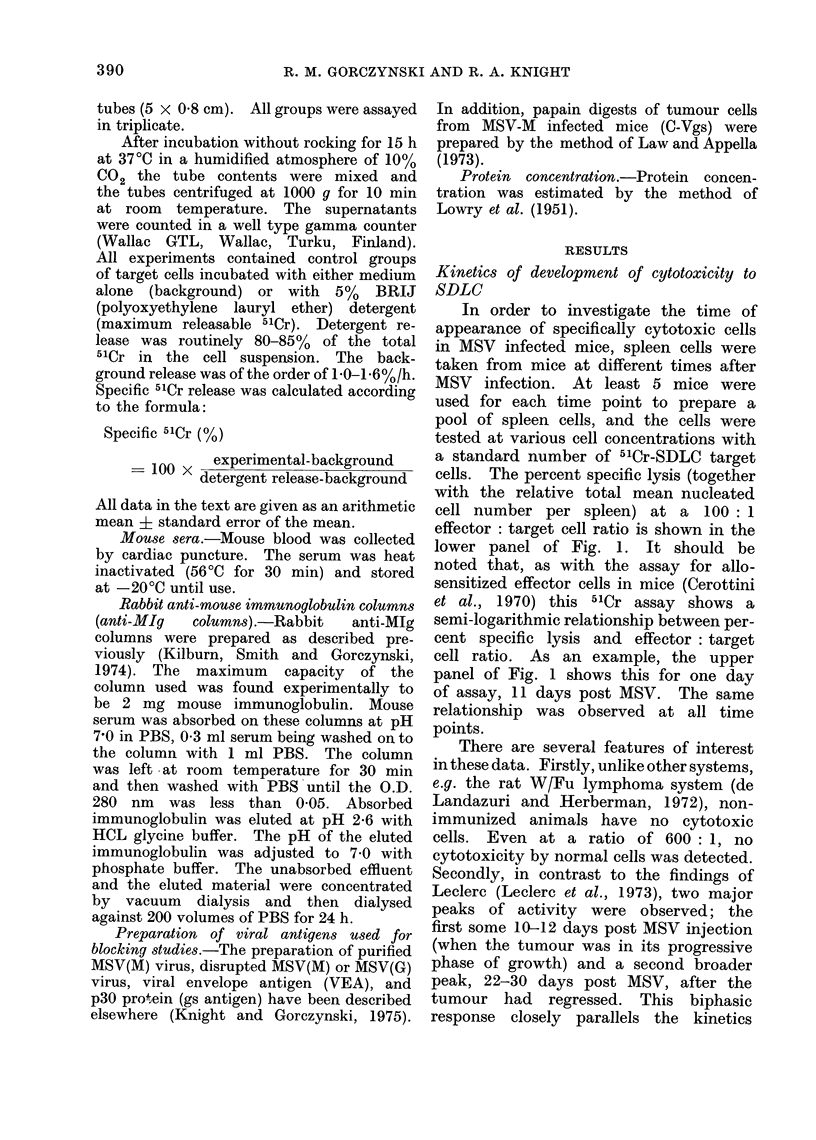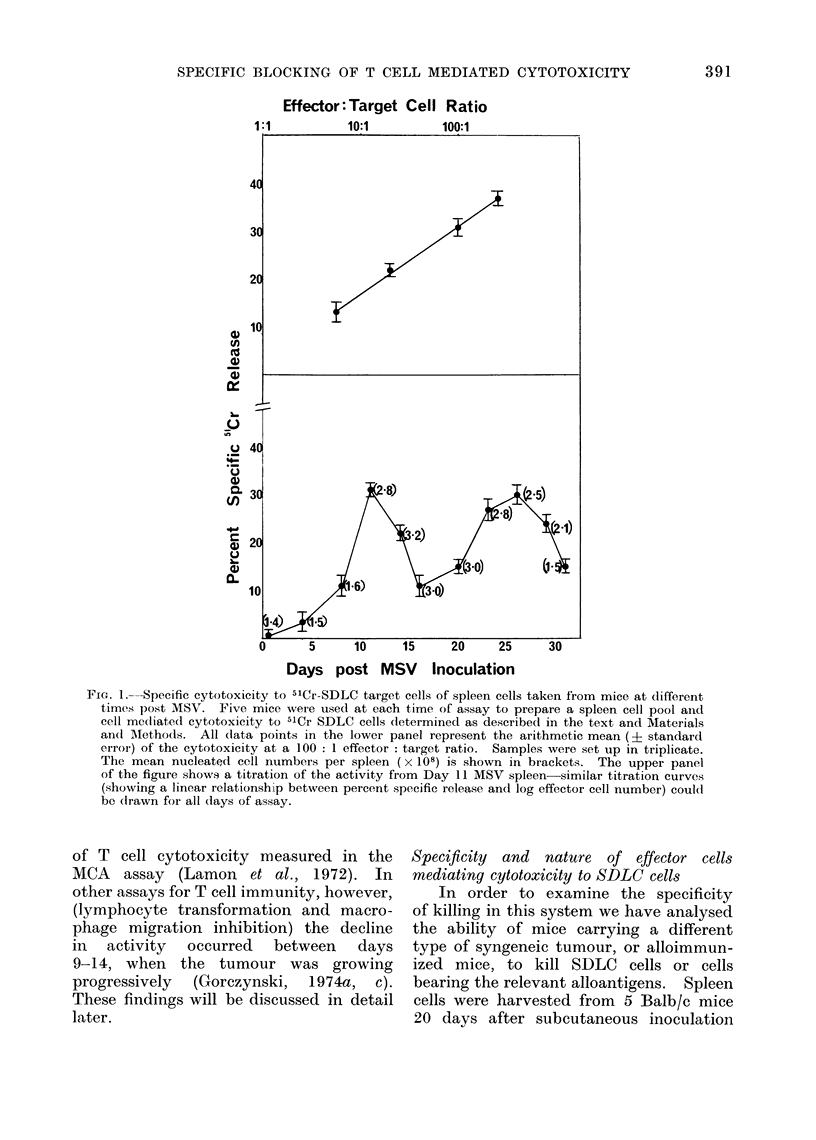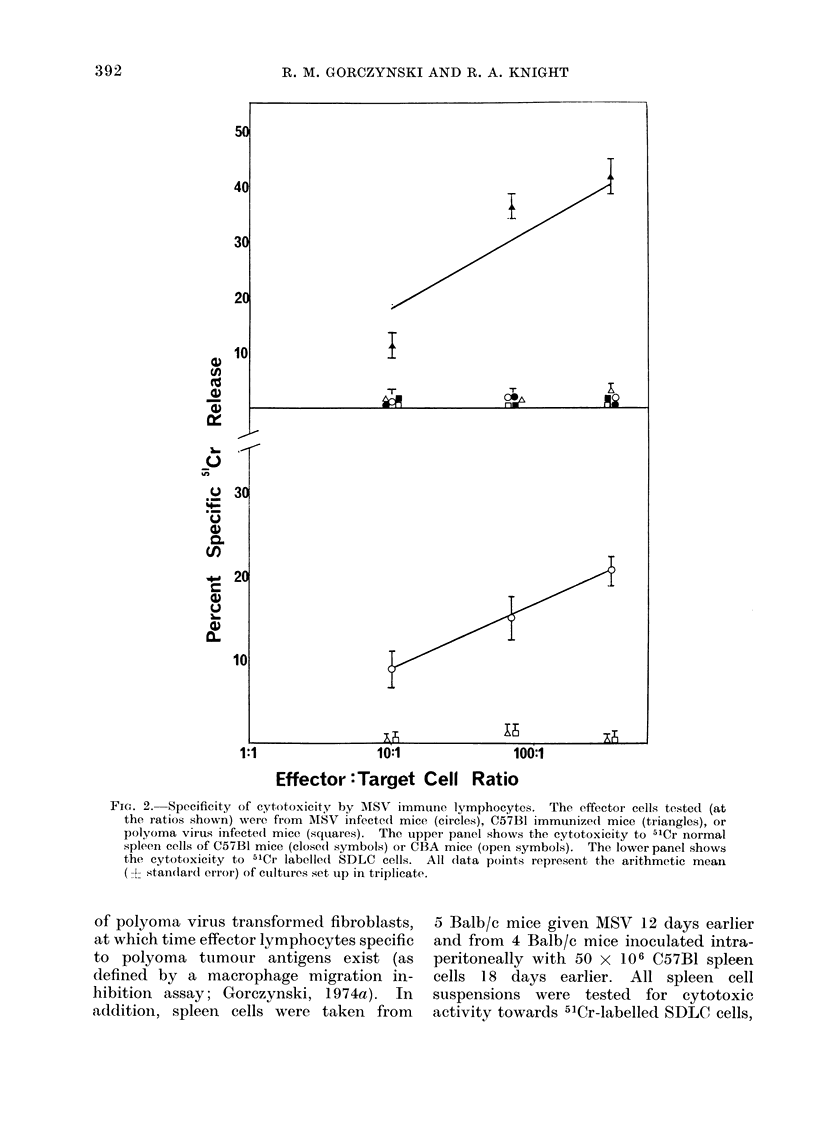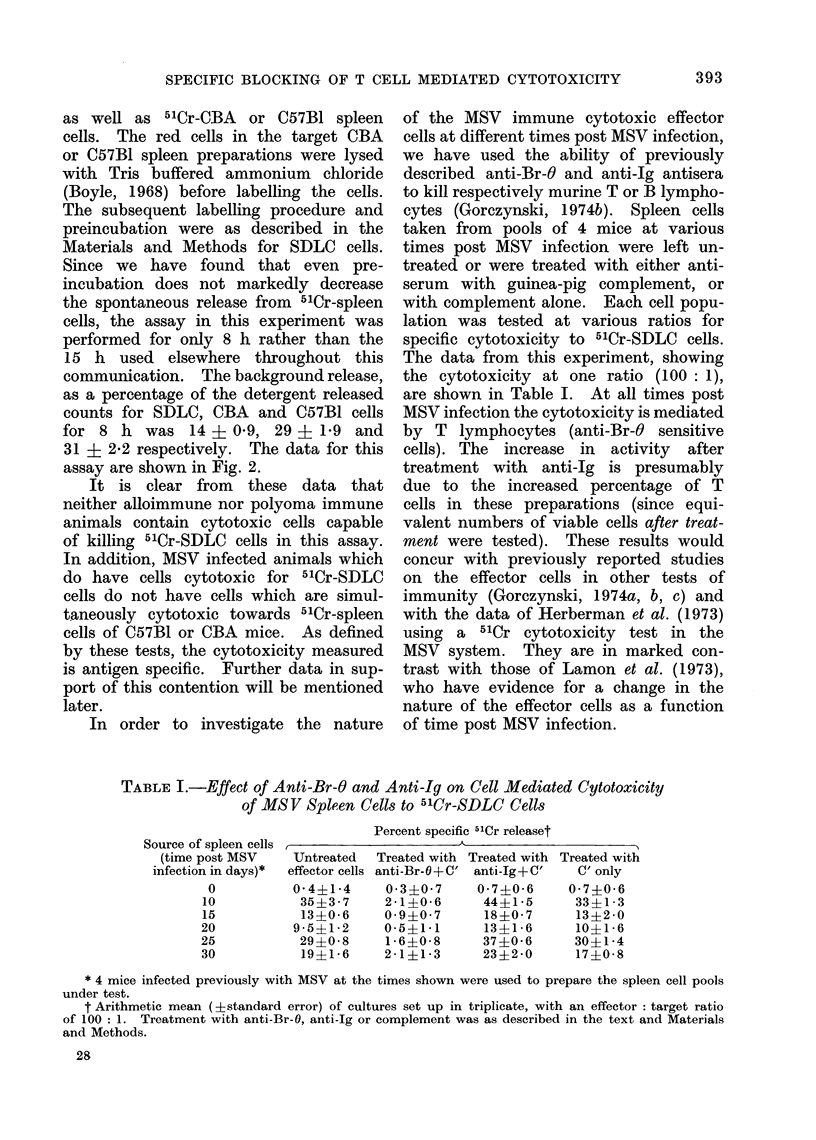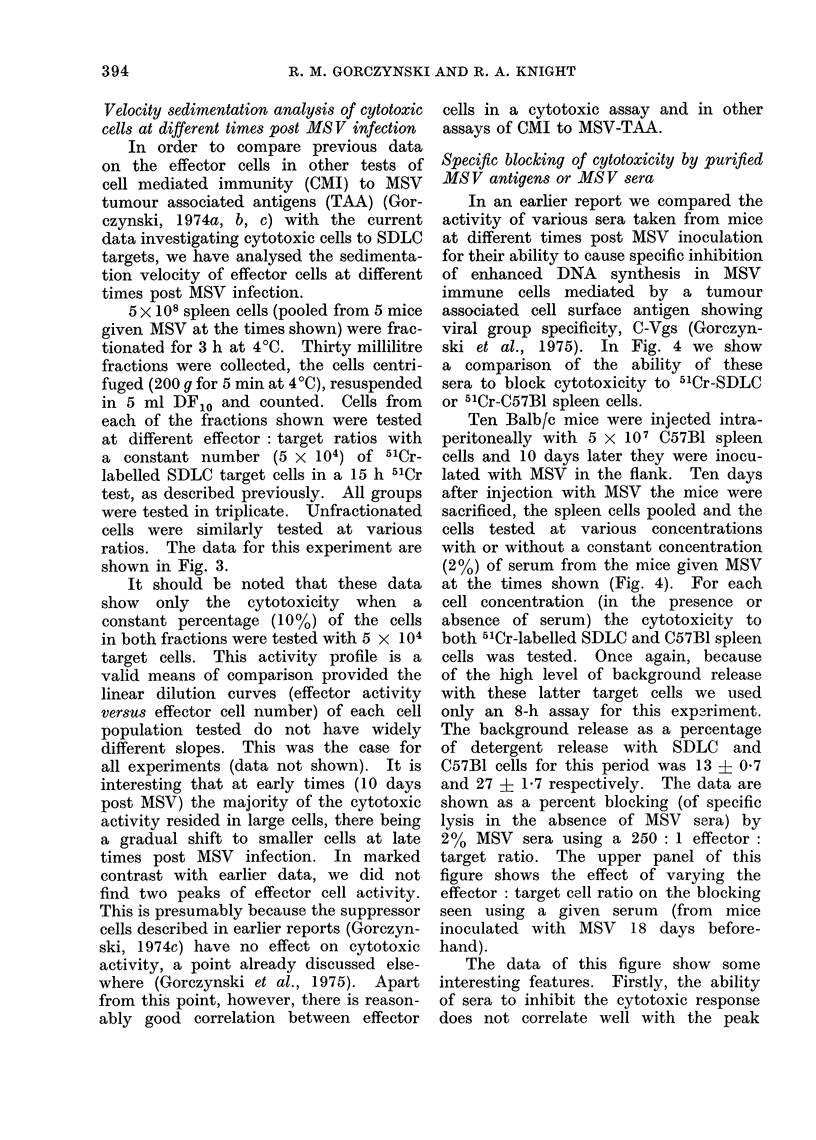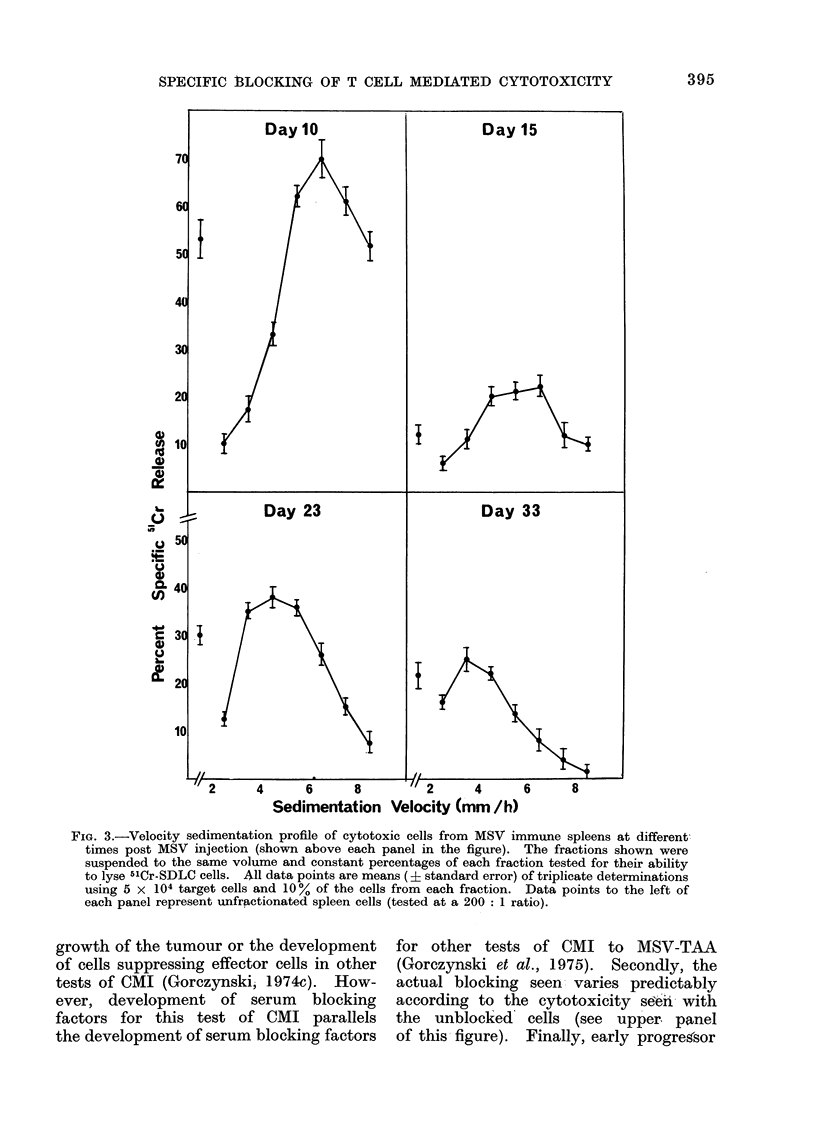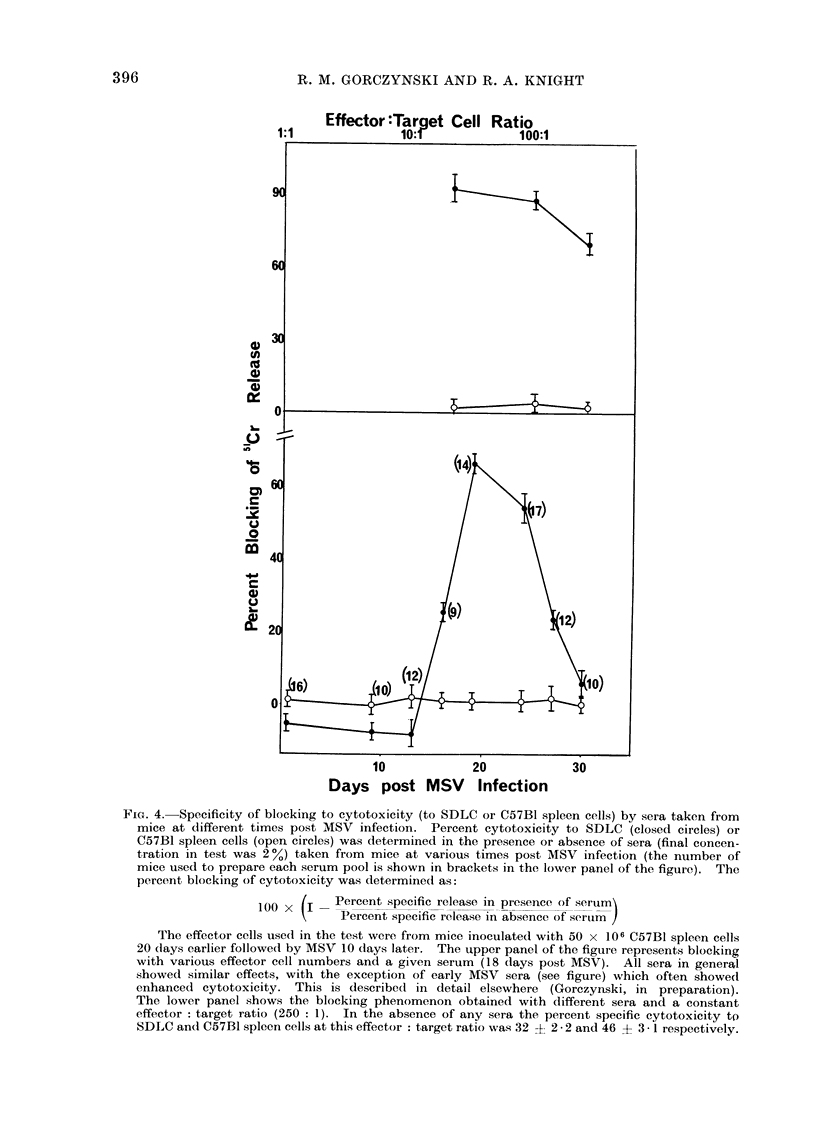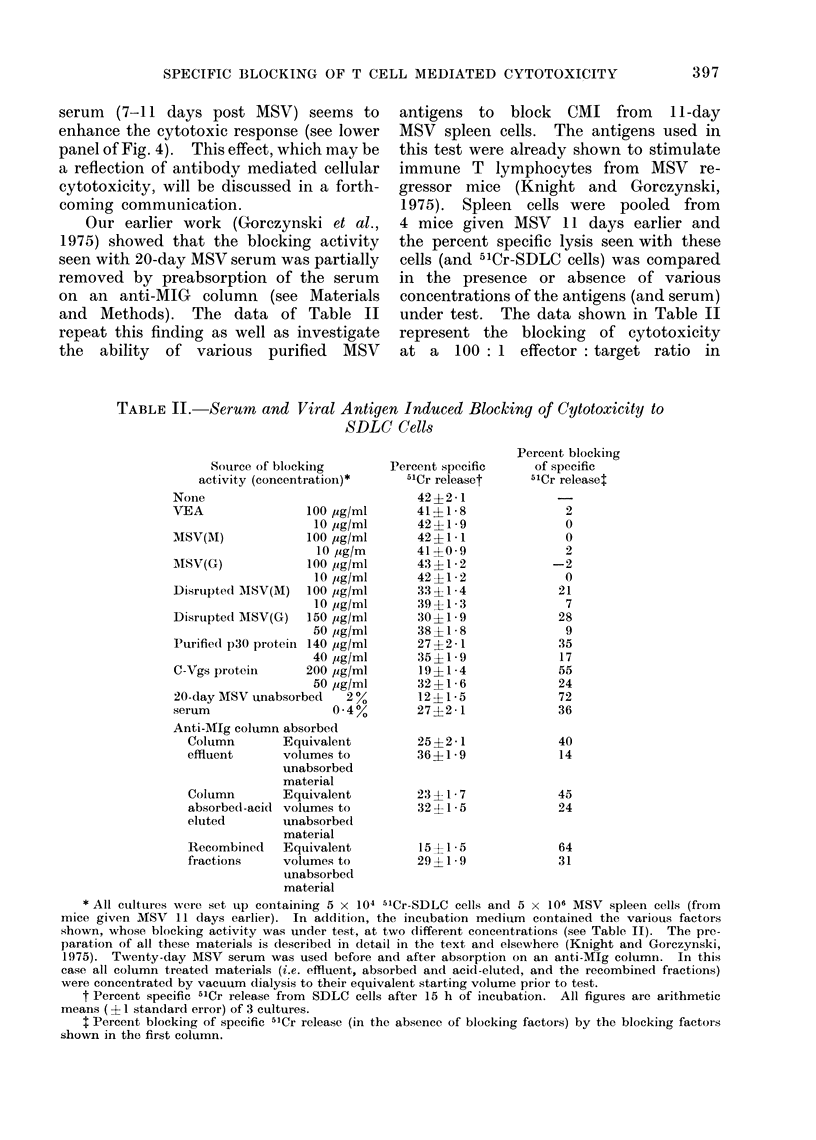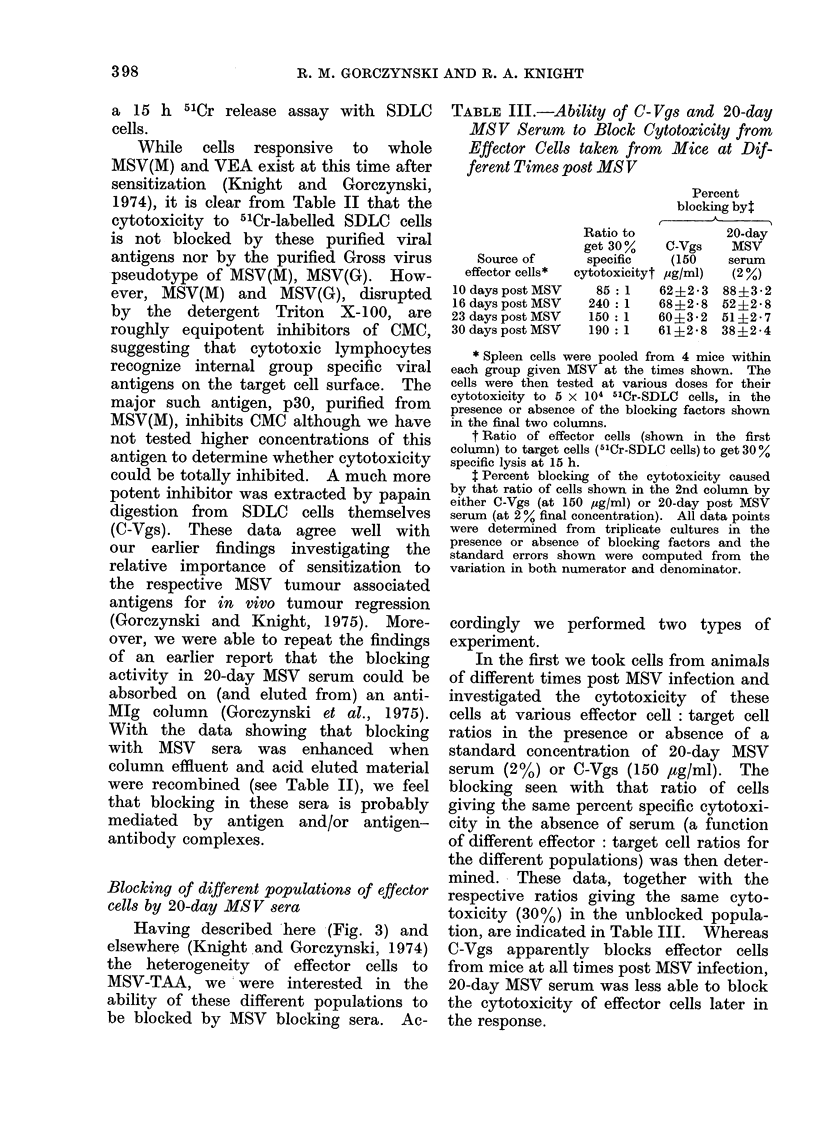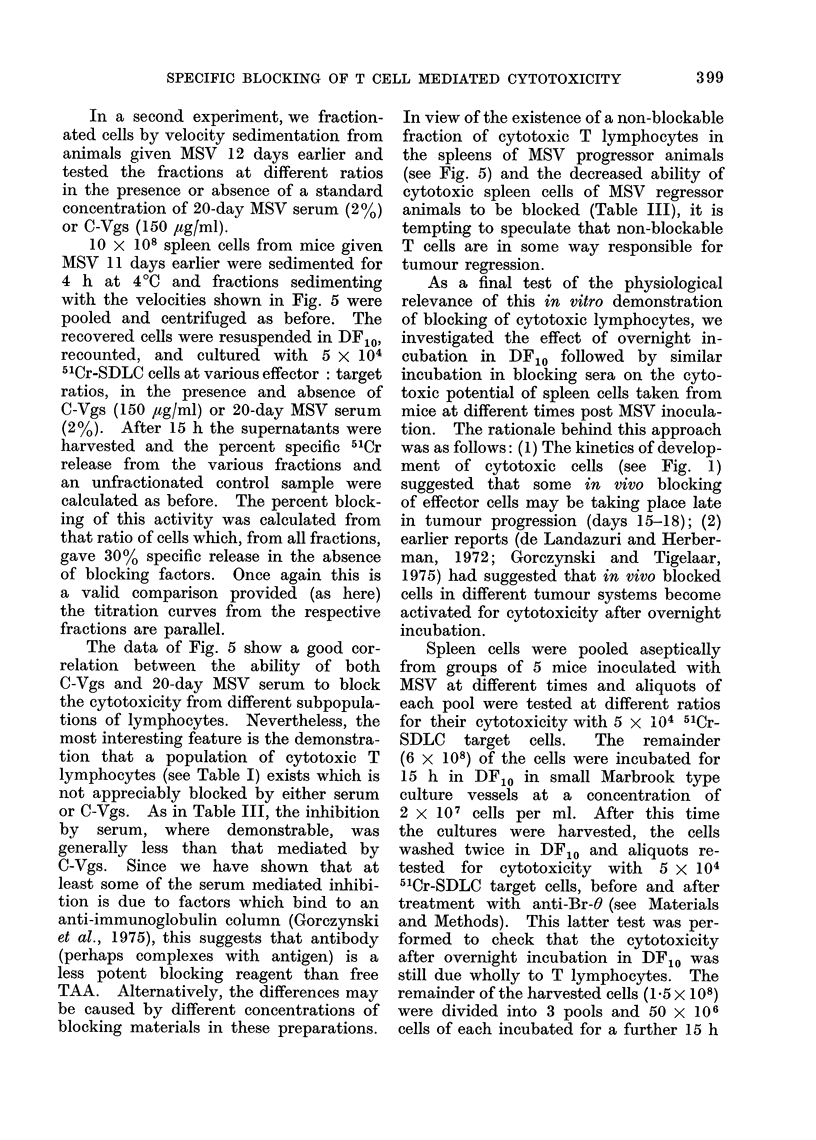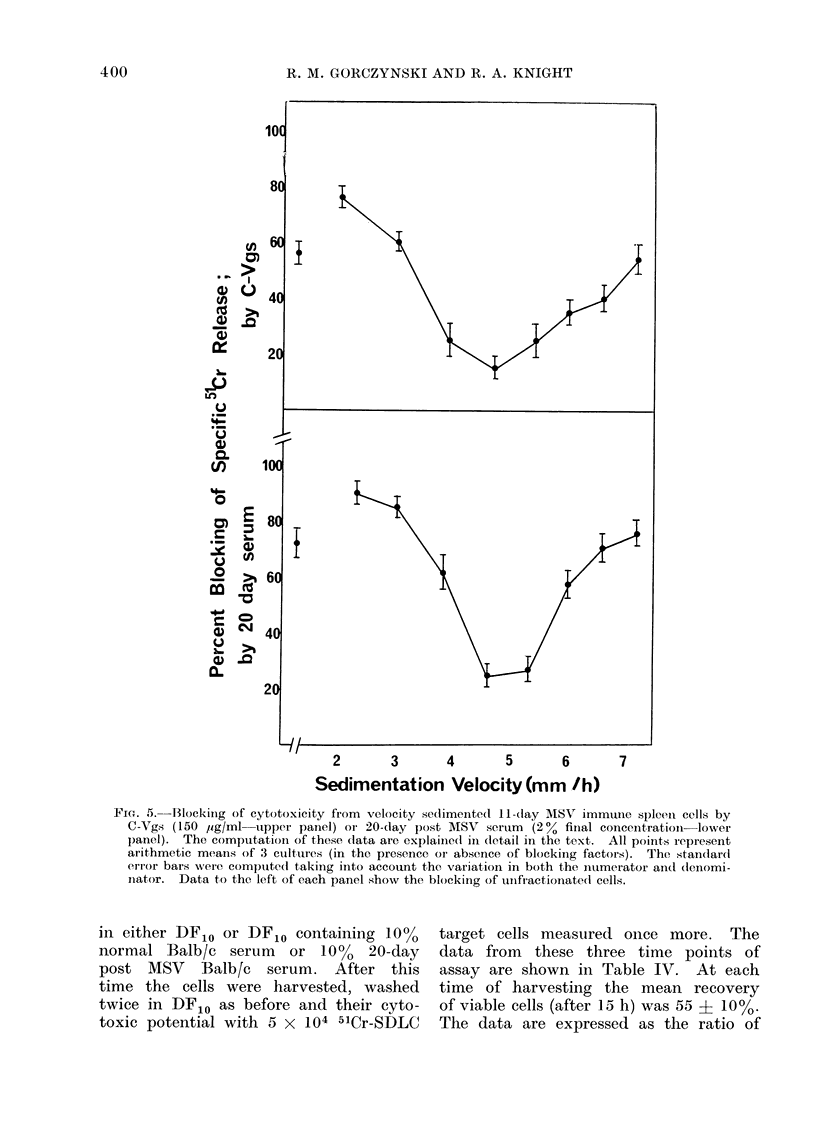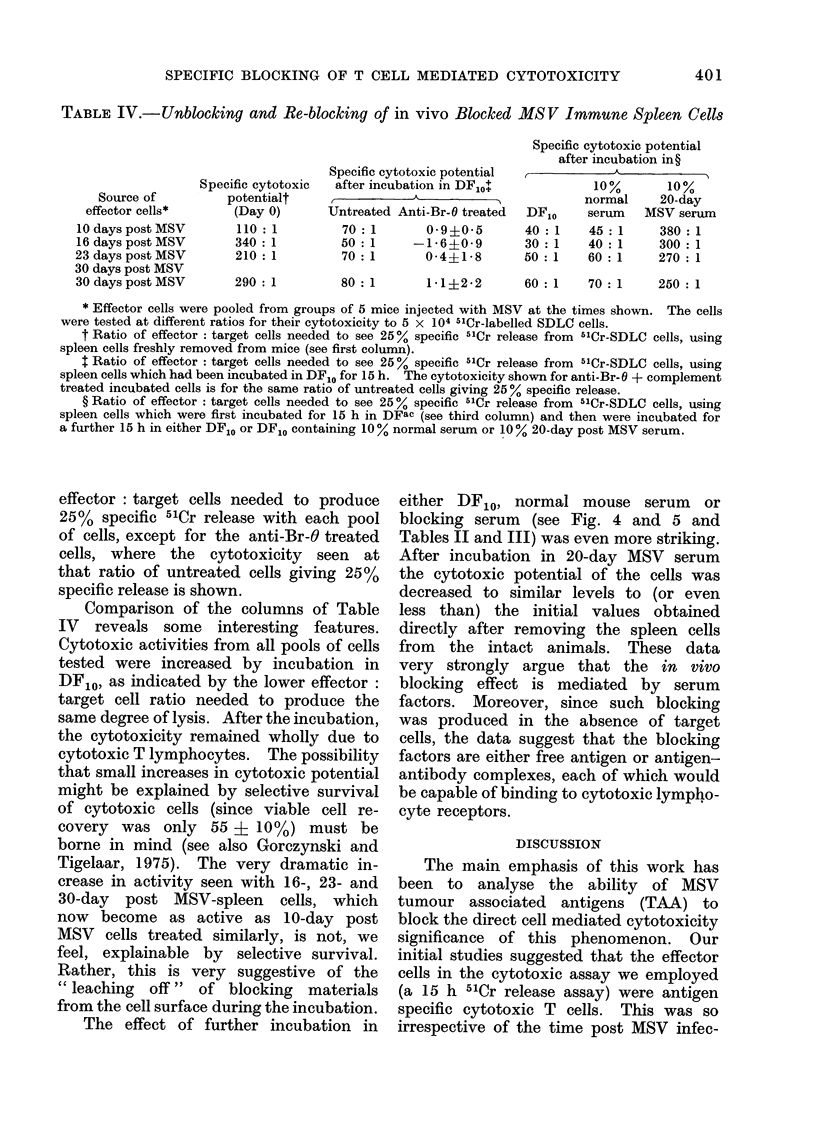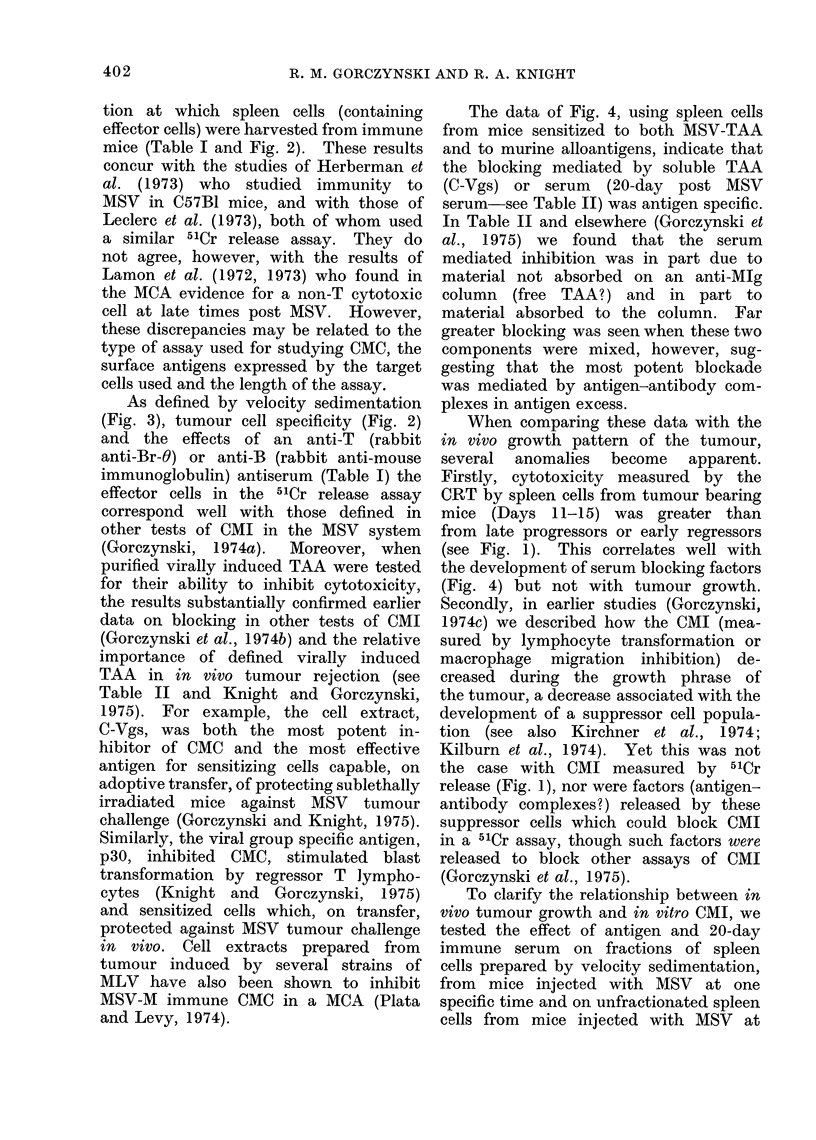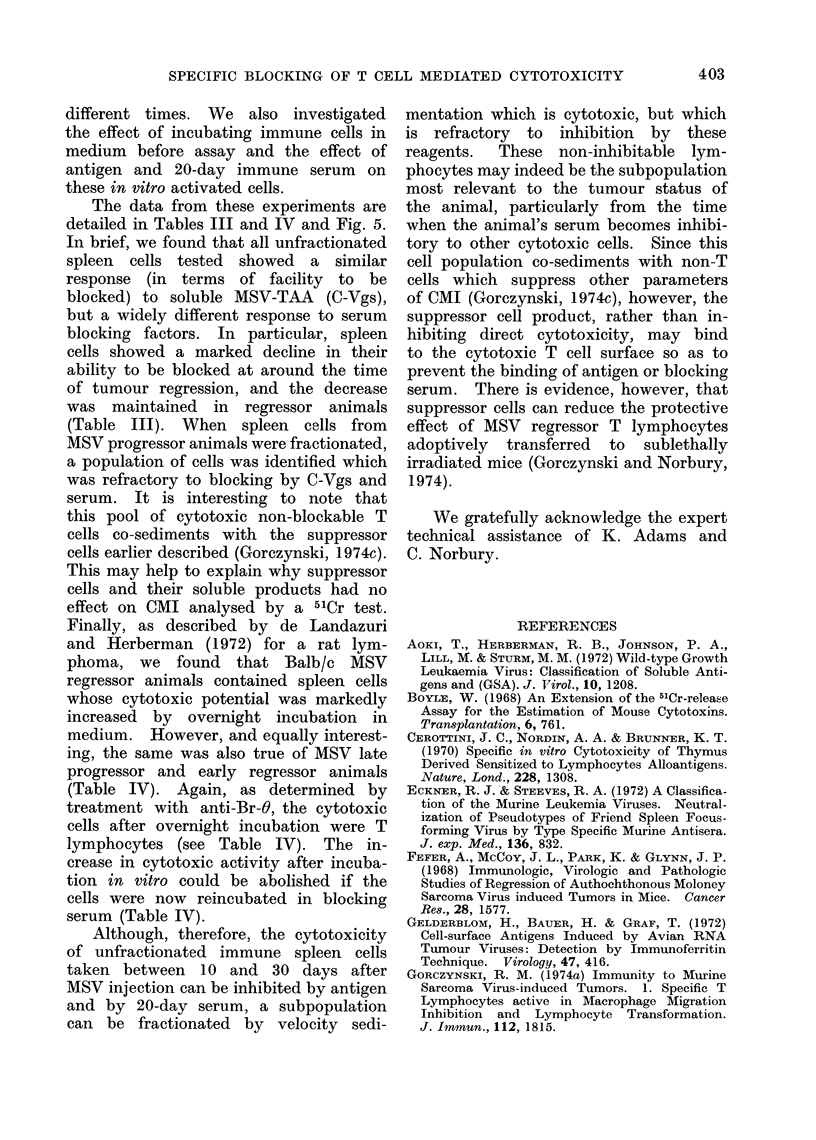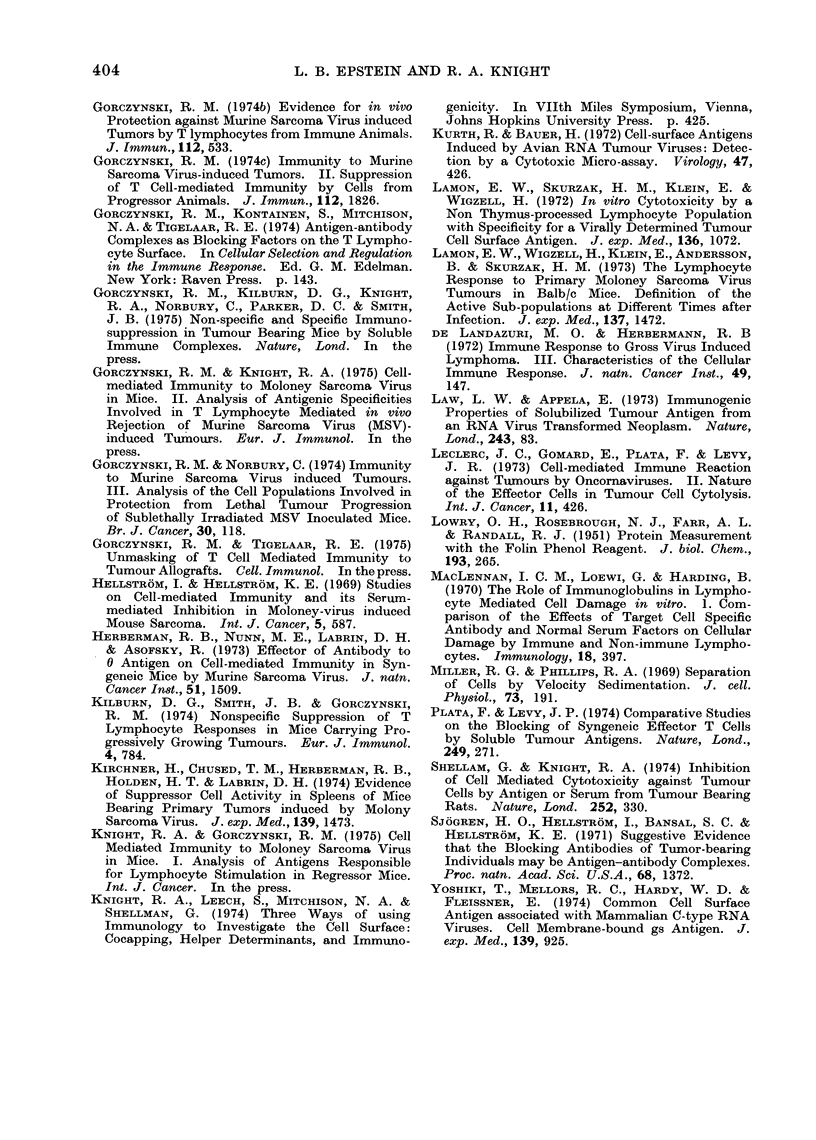# Immunity to murine sarcoma virus induced tumours. IV. Direct cellular cytolysis of 51Cr labelled target cells in vitro and analysis of blocking factors which modulate cytotoxicity.

**DOI:** 10.1038/bjc.1975.78

**Published:** 1975-04

**Authors:** R. M. Gorczynski, R. A. Knight

## Abstract

The antigen specific cell mediated cytotoxicity of MSV immune spleen lymphocytes to 51Cr labelled murine lymphoma cells was wholly abolished by pretreatment of the spleen cells with anti-theta antibody and complement. Early during the immune response to MSV the cytotoxic acitivity was inhibited by incubation of immune lymphocytes with "late progressor" or "early regressor" serum. Immune lymphocytes at later times were more refractory to such inhibition by serum blocking factors. Although unfractionated cytotoxic lymphocytes, irrespective of the time after MSV infection at which they were tested, were inhibited by soluble tumour associated antigen (TAA), a subpopulation of cytotoxic T cells was identified which was inhibited neither by antigen nor serum.


*Br. J. Cancer* (1975) **31**, 387

### IMMUNITY TO MURINE SARCOMA VIRUS INDUCED TUMOURS. IV. DIRECT CELLULAR CYTOLYSIS OF $^{51}Cr$ LABELLED TARGET CELLS *IN VITRO* AND ANALYSIS OF BLOCKING FACTORS WHICH MODULATE CYTOTOXICITY

R. M. GORCZYNSKI* AND R. A. KNIGHT†

*From the Imperial Cancer Research Fund, Tumour Immunology Unit, Department of Zoology, University College, London, Gower Street, London WC1E 6BT*

Received 17 December 1974. Accepted 23 December 1974

**Summary.—The antigen specific cell mediated cytotoxicity of MSV immune spleen lymphocytes to $^{51}Cr$ labelled murine lymphoma cells was wholly abolished by pretreatment of the spleen cells with anti-θ antibody and complement. Early during the immune response to MSV the cytotoxic activity was inhibited by incubation of immune lymphocytes with " late progressor " or " early regressor " serum. Immune lymphocytes at later times were more refractory to such inhibition by serum blocking factors.**

**Although unfractionated cytotoxic lymphocytes, irrespective of the time after MSV infection at which they were tested, were inhibited by soluble tumour associated antigen (TAA), a subpopulation of cytotoxic T cells was identified which was inhibited neither by antigen nor serum.**

THE NATURAL history and immune response to tumours induced by Moloney mouse sarcoma virus (MSV(MLV-M)) in Balb/c mice have been studied extensively. In adult animals, tumours generally develop at the site of injection within 5–8 days and reach a maximum size in about 10–15 days (Fefer *et al.*, 1968). Most tumours subsequently regress, the regression usually being complete by 25 days (Fefer *et al.*, 1968; Lamon *et al.*, 1972).

Several workers have demonstrated syngeneic cell mediated cytotoxicity (CMC) in this system (Hellström and Hellström, 1969; Lamon *et al.*, 1973; Leclerc *et al.*, 1973; Plata and Levy, 1974). CMC by both T and non-T lymphocytes has been detected in a microcytotoxicity (MCA) assay (Lamon *et al.*, 1973). Cytotoxic T cells were found immediately before tumour development and during regression, but disappeared after 30 days. The cytotoxic non-T cell population, however, although also present before the tumour had appeared and during regression, was most active at 30 days and was still active at Day 60. Neither T nor non-T cells were active at the time of peak tumour size (Lamon *et al.*, 1973).

In contrast, the peak cytolytic activity in a chromium release test (CRT), which, as in allogeneic systems, measures exclusively T cell cytotoxicity was found at about Day 15 (Cerottini, Nordin and Brunner, 1970; Leclerc *et al.*, 1973). The differences in cytotoxic T cell kinetics in CRT and MCA have been confirmed using the same immune lymphoid cell preparations in both assays (Plata and Levy, 1974). In a similar rat lymphoma system, the cytotoxicity of immune T cells can be increased by incubation *in vitro* (de Landazuri and Herberman, 1972). Since the MCA measures a long-

Supported by: * Imperial Cancer Research Fund. Present address, Ontario Cancer Institute, Toronto M4X 1K9, Canada. † Medical Research Council of Great Britain.

term effect, in contrast to the short term CRT, the apparent spontaneous activity of immune T cells in this assay may merely reflect the potential of cells activated *in vitro*. Furthermore, since killing by non-T cells in the MCA does not occur in the absence of B cells (Lamon *et al.*, 1973), this effect may be mediated by Fc receptor bearing non-immune cells in the presence of sensitizing antibody (Maclennan, Loewi and Harding, 1970). Immune T lymphocytes, however, are specifically sensitized to tumour associated antigens in MSV regressor mice (Gorczynski, 1974*a*; Knight and Gorczynski, 1975), and these immune T cells can aid in tumour rejection (Gorczynski, 1974*b*; Gorczynski and Norbury, 1974).

Of equal interest to the question of the nature of the immunologically reactive cells in tumour bearing (and regressor) mice, is the question of the role of specific and nonspecific immunosuppressing factors in these animals. Earlier studies in the MSV system suggested that progressor animals contained factors in the serum able to block the cytotoxicity *in vitro* of progressor and regressor lymphocytes (Hellström and Hellström, 1969). Evidence was presented that such blocking factors in tumour bearer animals were antigen–antibody complexes (Sjögren *et al.*, 1971). Using a more quantitative $^{51}$Cr cytotoxicity assay, Leclerc *et al.* (1973) were unable to reproduce these findings. Moreover, it has recently become apparent that progressor animals contain a cell population able to block the *in vitro* activity of T lymphocytes in a nonspecific manner (Gorczynski, 1974*c*). It has been suggested that this type of effect could still be mediated *via* antigen–antibody complexes (Gorczynski *et al.*, 1974) and evidence to support this contention has recently been presented (Gorczynski *et al.*, 1975). There is at present only circumstantial evidence that specific and nonspecific blocking factors analysed *in vitro* have any relevance to the *in vivo* tumour situation.

One other area which we at present know little about is the role of virus coded antigens in the immune response to virally induced tumours. There is now evidence that both virus coded antigens (*e.g.* viral envelope antigen, VEA) and a virus induced antigen with viral group specificity exist as tumour associated surface antigens (TASA) in the avian RNA tumour virus situation (Gelderblom, Bauer and Graf, 1972) and in the MSV tumour situation (Eckner and Steeves, 1972; Aoki *et al.*, 1972; Yoshiki *et al.*, 1974). Lymphocytes reactive to the two avian TASA (Kurth and Bauer, 1972) have been detected by microcytotoxicity tests. In a rat MLV-G induced lymphoma system, cytotoxic antibody is directed principally, and immune lymphocytes partially, against the internal virion antigen, p30 (Shellam and Knight, 1974; Knight *et al.*, 1974). In the murine MSV system there is evidence that p30 is the main tumour rejection antigen (Gorczynski and Knight, 1975). Furthermore, lymphocytes reactive both to this antigen and to the viral envelope glycoprotein antigens (VEA) have been detected by lymphocyte transformation tests (Knight and Gorczynski, 1975).

We have developed a $^{51}$Cr cytotoxicity assay for lymphoid cells from MSV inoculated mice with a view to answering several questions of fundamental importance. We wished to examine the kinetics of the development of cell mediated cytotoxicity, the cell(s) responsible for this lysis and the relationship between these cell types and those described using alternative assays for immunity in this system. Finally, we have examined the ability of purified viral antigens and serum taken from mice at different times post MSV inoculation to block direct cell mediated cytotoxicity caused by different populations of effector cells.

### MATERIALS AND METHODS

*Mice.*—Male Balb/c and C57Bl/Blue (hereafter referred to as C57Bl for clarity) were obtained from the ICRF breeding unit at Mill Hill.

*Tumours and* in vivo *sensitization.*—Sarcomata were induced in 30-day old Balb/c mice by injection in the thigh of 0·1 ml crude MSV tumour homogenate as described earlier (Gorczynski, 1974*a*). An MSV derived lymphoma was obtained by passaging MSV transformed fibroblasts once through young Balb/c mice before establishing the transformed cells as an *in vitro* suspension culture. The medium used for such cultures was Dulbecco's modified Eagle's medium (with added glutamine, penicillin and streptomycin) supplemented with 10% foetal calf serum, $DF_{10}$. The cells were cultured in $DF_{10}$ and grew with a doubling time of 8–10 h provided the concentration was kept below $1 \times 10^6$/ml. The harvested cells provided excellent reproducible targets for the cytotoxic assay (see below). This cell line (subsequently referred to as SDLC) grew *in vivo* in Balb/c as an ascitic tumour. Balb/c mice were sensitized to C57Bl alloantigens by inoculation of $50 \times 10^6$ C57Bl spleen cells intraperitoneally 18–20 days before sacrifice of the Balb/c.

*Cell preparation.*—Spleen cells were prepared by teasing the organs apart in ice-cold phosphate buffered saline (PBS). Cell clumps were allowed to settle out for 10 min at 4°C and the cells centrifuged at 200 *g* for 5 min at 4°C. The cells were then resuspended in PBS with 0·1% bovine serum albumin (BSA) or $DF_{10}$, depending on the subsequent manipulations to be performed with them. Unless otherwise stated, all cell concentrations refer to viable nucleated cells determined by trypan blue dye exclusion.

*Velocity sedimentation cell separation.*—This technique, which separates cells primarily according to their size, has been described elsewhere (Miller and Phillips, 1969). Sterile glass chambers, either 11·0 cm or 16·8 cm in diameter (Aimer Glass Co., London, England) were used according to the number of cells to be separated. The initial cell band was loaded in 0·3% BSA in PBS, the total cell concentration (viable and non-viable) being no greater than $10 \times 10^6$/ml.

*Antisera and treatment of cells with antisera.*—The preparation of 2 rabbit antisera, anti-mouse brain associated theta (anti-Br-$\theta$) and anti-immunoglobulin (anti-Ig) are described in detail elsewhere (Gorczynski, 1974*b*). Also in this report are data confirming the specificity of these 2 antisera, in the presence of guinea-pig complement, for cytotoxicity to murine T or B lymphocytes respectively. Thus, the anti-Br-$\theta$ killed 70% of murine peripheral lymph node cells, 100% of mouse thymocytes and removed the PHA responsive cells (but not the LPS responsive cells) from a mouse spleen cell suspension. In addition, the anti-Br-$\theta$ removed the T helper cell activity for an anti-sheep erythrocyte (SRBC) antibody response while not affecting the B cell response to a thymus independent antigen (polymerized flagellin, POL). In contrast, the anti-Ig did not kill appreciable numbers of thymocytes or remove the PHA responsive cells from a mouse spleen cell suspension, or affect T helper cells for an anti-SRBC response. However, the anti-Ig did remove LPS responsive cells from a spleen cell suspension and did remove B cells responding to both thymus dependent (SRBC) and thymus independent (POL) antigens.

Cells to be treated with either antiserum were suspended in 0·1% BSA in PBS, with the final concentration of anti-serum present being 1/15 (anti-Br-$\theta$) or 1/10 (anti-Ig). The cells were incubated for 90 min at 4°C, washed (200 *g* for 5 min at 4°C), resuspended in either $DF_{10}$ or guinea-pig complement (diluted 1/10 in $DF_{10}$), and incubated for a further 45 min at 37°C. The cells were then rewashed as above and used as described in the text.

*Cytotoxicity assay.*—5 to $10 \times 10^6$ SDLC cells were radiolabelled in 1 ml $DF_{10}$ containing 100$\mu$Ci $^{51}Cr$ (as sodium chromate, Radiochemical Centre, Amersham, England) for 60 min at 37°C in 10% $CO_2$ in air. The cells were washed 4 times in 25 ml $DF_{10}$ (at room temperature). They were then resuspended in 25 ml $DF_{10}$ and incubated at 37°C in 10% $CO_2$ in air for a further 3–4 h before use as targets in the cytotoxic test. We found that this preincubation of the labelled target cells dramatically reduced their spontaneous release during the assay period and allowed the latter to be performed for 15 h with acceptably low spontaneous $^{51}Cr$ release. Immediately before use in the assay the cells were washed once as above and suspended to a concentration of $5 \times 10^5$/ml. $5 \times 10^4$ $^{51}Cr$ labelled SDLC cells were then mixed with varying numbers of the effector cells under test in 1 ml $DF_{10}$, in glass bacteriological test tubes (5 × 0·8 cm). All groups were assayed in triplicate.

After incubation without rocking for 15 h at 37°C in a humidified atmosphere of 10% $CO_2$ the tube contents were mixed and the tubes centrifuged at 1000 *g* for 10 min at room temperature. The supernatants were counted in a well type gamma counter (Wallac GTL, Wallac, Turku, Finland). All experiments contained control groups of target cells incubated with either medium alone (background) or with 5% BRIJ (polyoxyethylene lauryl ether) detergent (maximum releasable $^{51}$Cr). Detergent release was routinely 80–85% of the total $^{51}$Cr in the cell suspension. The background release was of the order of 1·0–1·6%/h. Specific $^{51}$Cr release was calculated according to the formula:

$$\text{Specific } ^{51}\text{Cr } (\%) = 100 \times \frac{\text{experimental-background}}{\text{detergent release-background}}$$

All data in the text are given as an arithmetic mean ± standard error of the mean.

*Mouse sera*.—Mouse blood was collected by cardiac puncture. The serum was heat inactivated (56°C for 30 min) and stored at −20°C until use.

*Rabbit anti-mouse immunoglobulin columns* (*anti-MIg columns*).—Rabbit anti-MIg columns were prepared as described previously (Kilburn, Smith and Gorczynski, 1974). The maximum capacity of the column used was found experimentally to be 2 mg mouse immunoglobulin. Mouse serum was absorbed on these columns at pH 7·0 in PBS, 0·3 ml serum being washed on to the column with 1 ml PBS. The column was left at room temperature for 30 min and then washed with PBS until the O.D. 280 nm was less than 0·05. Absorbed immunoglobulin was eluted at pH 2·6 with HCL glycine buffer. The pH of the eluted immunoglobulin was adjusted to 7·0 with phosphate buffer. The unabsorbed effluent and the eluted material were concentrated by vacuum dialysis and then dialysed against 200 volumes of PBS for 24 h.

*Preparation of viral antigens used for blocking studies*.—The preparation of purified MSV(M) virus, disrupted MSV(M) or MSV(G) virus, viral envelope antigen (VEA), and p30 protein (gs antigen) have been described elsewhere (Knight and Gorczynski, 1975). In addition, papain digests of tumour cells from MSV-M infected mice (C-Vgs) were prepared by the method of Law and Appella (1973).

*Protein concentration*.—Protein concentration was estimated by the method of Lowry *et al.* (1951).

### RESULTS

### *Kinetics of development of cytotoxicity to SDLC*

In order to investigate the time of appearance of specifically cytotoxic cells in MSV infected mice, spleen cells were taken from mice at different times after MSV infection. At least 5 mice were used for each time point to prepare a pool of spleen cells, and the cells were tested at various cell concentrations with a standard number of $^{51}$Cr-SDLC target cells. The percent specific lysis (together with the relative total mean nucleated cell number per spleen) at a 100 : 1 effector : target cell ratio is shown in the lower panel of Fig. 1. It should be noted that, as with the assay for allosensitized effector cells in mice (Cerottini *et al.*, 1970) this $^{51}$Cr assay shows a semi-logarithmic relationship between percent specific lysis and effector : target cell ratio. As an example, the upper panel of Fig. 1 shows this for one day of assay, 11 days post MSV. The same relationship was observed at all time points.

There are several features of interest in these data. Firstly, unlike other systems, *e.g.* the rat W/Fu lymphoma system (de Landazuri and Herberman, 1972), non-immunized animals have no cytotoxic cells. Even at a ratio of 600 : 1, no cytotoxicity by normal cells was detected. Secondly, in contrast to the findings of Leclerc (Leclerc *et al.*, 1973), two major peaks of activity were observed; the first some 10–12 days post MSV injection (when the tumour was in its progressive phase of growth) and a second broader peak, 22–30 days post MSV, after the tumour had regressed. This biphasic response closely parallels the kinetics

FIG. 1.—Specific cytotoxicity to $^{51}$Cr-SDLC target cells of spleen cells taken from mice at different times post MSV. Five mice were used at each time of assay to prepare a spleen cell pool and cell mediated cytotoxicity to $^{51}$Cr SDLC cells determined as described in the text and Materials and Methods. All data points in the lower panel represent the arithmetic mean ($\pm$ standard error) of the cytotoxicity at a 100 : 1 effector : target ratio. Samples were set up in triplicate. The mean nucleated cell numbers per spleen ($\times 10^8$) is shown in brackets. The upper panel of the figure shows a titration of the activity from Day 11 MSV spleen—similar titration curves (showing a linear relationship between percent specific release and log effector cell number) could be drawn for all days of assay.

of T cell cytotoxicity measured in the MCA assay (Lamon *et al.*, 1972). In other assays for T cell immunity, however, (lymphocyte transformation and macrophage migration inhibition) the decline in activity occurred between days 9–14, when the tumour was growing progressively (Gorczynski, 1974*a*, *c*). These findings will be discussed in detail later.

*Specificity and nature of effector cells mediating cytotoxicity to SDLC cells*

In order to examine the specificity of killing in this system we have analysed the ability of mice carrying a different type of syngeneic tumour, or alloimmunized mice, to kill SDLC cells or cells bearing the relevant alloantigens. Spleen cells were harvested from 5 Balb/c mice 20 days after subcutaneous inoculation

FIG. 2.—Specificity of cytotoxicity by MSV immune lymphocytes. The effector cells tested (at the ratios shown) were from MSV infected mice (circles), C57Bl immunized mice (triangles), or polyoma virus infected mice (squares). The upper panel shows the cytotoxicity to $^{51}$Cr normal spleen cells of C57Bl mice (closed symbols) or CBA mice (open symbols). The lower panel shows the cytotoxicity to $^{51}$Cr labelled SDLC cells. All data points represent the arithmetic mean (± standard error) of cultures set up in triplicate.

of polyoma virus transformed fibroblasts, at which time effector lymphocytes specific to polyoma tumour antigens exist (as defined by a macrophage migration inhibition assay; Gorczynski, 1974*a*). In addition, spleen cells were taken from 5 Balb/c mice given MSV 12 days earlier and from 4 Balb/c mice inoculated intraperitoneally with 50 × $10^6$ C57Bl spleen cells 18 days earlier. All spleen cell suspensions were tested for cytotoxic activity towards $^{51}$Cr-labelled SDLC cells, as well as $^{51}Cr$-CBA or C57Bl spleen cells. The red cells in the target CBA or C57Bl spleen preparations were lysed with Tris buffered ammonium chloride (Boyle, 1968) before labelling the cells. The subsequent labelling procedure and preincubation were as described in the Materials and Methods for SDLC cells. Since we have found that even preincubation does not markedly decrease the spontaneous release from $^{51}Cr$-spleen cells, the assay in this experiment was performed for only 8 h rather than the 15 h used elsewhere throughout this communication. The background release, as a percentage of the detergent released counts for SDLC, CBA and C57Bl cells for 8 h was $14 \pm 0{\cdot}9$, $29 \pm 1{\cdot}9$ and $31 \pm 2{\cdot}2$ respectively. The data for this assay are shown in Fig. 2.

It is clear from these data that neither alloimmune nor polyoma immune animals contain cytotoxic cells capable of killing $^{51}Cr$-SDLC cells in this assay. In addition, MSV infected animals which do have cells cytotoxic for $^{51}Cr$-SDLC cells do not have cells which are simultaneously cytotoxic towards $^{51}Cr$-spleen cells of C57Bl or CBA mice. As defined by these tests, the cytotoxicity measured is antigen specific. Further data in support of this contention will be mentioned later.

In order to investigate the nature of the MSV immune cytotoxic effector cells at different times post MSV infection, we have used the ability of previously described anti-Br-$\theta$ and anti-Ig antisera to kill respectively murine T or B lymphocytes (Gorczynski, 1974*b*). Spleen cells taken from pools of 4 mice at various times post MSV infection were left untreated or were treated with either antiserum with guinea-pig complement, or with complement alone. Each cell population was tested at various ratios for specific cytotoxicity to $^{51}Cr$-SDLC cells. The data from this experiment, showing the cytotoxicity at one ratio (100 : 1), are shown in Table I. At all times post MSV infection the cytotoxicity is mediated by T lymphocytes (anti-Br-$\theta$ sensitive cells). The increase in activity after treatment with anti-Ig is presumably due to the increased percentage of T cells in these preparations (since equivalent numbers of viable cells *after treatment* were tested). These results would concur with previously reported studies on the effector cells in other tests of immunity (Gorczynski, 1974*a*, *b*, *c*) and with the data of Herberman *et al.* (1973) using a $^{51}Cr$ cytotoxicity test in the MSV system. They are in marked contrast with those of Lamon *et al.* (1973), who have evidence for a change in the nature of the effector cells as a function of time post MSV infection.

TABLE I.—*Effect of Anti-Br-θ and Anti-Ig on Cell Mediated Cytotoxicity of MSV Spleen Cells to ⁵¹Cr-SDLC Cells*

| Source of spleen cells (time post MSV infection in days)* | Percent specific $^{51}Cr$ release† | | | |
|---|---|---|---|---|
| | Untreated effector cells | Treated with anti-Br-$\theta$+C′ | Treated with anti-Ig+C′ | Treated with C′ only |
| 0 | 0·4±1·4 | 0·3±0·7 | 0·7±0·6 | 0·7±0·6 |
| 10 | 35±3·7 | 2·1±0·6 | 44±1·5 | 33±1·3 |
| 15 | 13±0·6 | 0·9±0·7 | 18±0·7 | 13±2·0 |
| 20 | 9·5±1·2 | 0·5±1·1 | 13±1·6 | 10±1·6 |
| 25 | 29±0·8 | 1·6±0·8 | 37±0·6 | 30±1·4 |
| 30 | 19±1·6 | 2·1±1·3 | 23±2·0 | 17±0·8 |

* 4 mice infected previously with MSV at the times shown were used to prepare the spleen cell pools under test.

† Arithmetic mean (±standard error) of cultures set up in triplicate, with an effector : target ratio of 100 : 1. Treatment with anti-Br-$\theta$, anti-Ig or complement was as described in the text and Materials and Methods.

*Velocity sedimentation analysis of cytotoxic cells at different times post MSV infection*

In order to compare previous data on the effector cells in other tests of cell mediated immunity (CMI) to MSV tumour associated antigens (TAA) (Gorczynski, 1974*a*, *b*, *c*) with the current data investigating cytotoxic cells to SDLC targets, we have analysed the sedimentation velocity of effector cells at different times post MSV infection.

$5 \times 10^8$ spleen cells (pooled from 5 mice given MSV at the times shown) were fractionated for 3 h at 4°C. Thirty millilitre fractions were collected, the cells centrifuged (200 *g* for 5 min at 4°C), resuspended in 5 ml $DF_{10}$ and counted. Cells from each of the fractions shown were tested at different effector : target ratios with a constant number ($5 \times 10^4$) of $^{51}$Cr-labelled SDLC target cells in a 15 h $^{51}$Cr test, as described previously. All groups were tested in triplicate. Unfractionated cells were similarly tested at various ratios. The data for this experiment are shown in Fig. 3.

It should be noted that these data show only the cytotoxicity when a constant percentage (10%) of the cells in both fractions were tested with $5 \times 10^4$ target cells. This activity profile is a valid means of comparison provided the linear dilution curves (effector activity *versus* effector cell number) of each cell population tested do not have widely different slopes. This was the case for all experiments (data not shown). It is interesting that at early times (10 days post MSV) the majority of the cytotoxic activity resided in large cells, there being a gradual shift to smaller cells at late times post MSV infection. In marked contrast with earlier data, we did not find two peaks of effector cell activity. This is presumably because the suppressor cells described in earlier reports (Gorczynski, 1974*c*) have no effect on cytotoxic activity, a point already discussed elsewhere (Gorczynski *et al.*, 1975). Apart from this point, however, there is reasonably good correlation between effector cells in a cytotoxic assay and in other assays of CMI to MSV-TAA.

*Specific blocking of cytotoxicity by purified MSV antigens or MSV sera*

In an earlier report we compared the activity of various sera taken from mice at different times post MSV inoculation for their ability to cause specific inhibition of enhanced DNA synthesis in MSV immune cells mediated by a tumour associated cell surface antigen showing viral group specificity, C-Vgs (Gorczynski *et al.*, 1975). In Fig. 4 we show a comparison of the ability of these sera to block cytotoxicity to $^{51}$Cr-SDLC or $^{51}$Cr-C57Bl spleen cells.

Ten Balb/c mice were injected intraperitoneally with $5 \times 10^7$ C57Bl spleen cells and 10 days later they were inoculated with MSV in the flank. Ten days after injection with MSV the mice were sacrificed, the spleen cells pooled and the cells tested at various concentrations with or without a constant concentration (2%) of serum from the mice given MSV at the times shown (Fig. 4). For each cell concentration (in the presence or absence of serum) the cytotoxicity to both $^{51}$Cr-labelled SDLC and C57Bl spleen cells was tested. Once again, because of the high level of background release with these latter target cells we used only an 8-h assay for this experiment. The background release as a percentage of detergent release with SDLC and C57Bl cells for this period was $13 \pm 0{\cdot}7$ and $27 \pm 1{\cdot}7$ respectively. The data are shown as a percent blocking (of specific lysis in the absence of MSV sera) by 2% MSV sera using a 250 : 1 effector : target ratio. The upper panel of this figure shows the effect of varying the effector : target cell ratio on the blocking seen using a given serum (from mice inoculated with MSV 18 days beforehand).

The data of this figure show some interesting features. Firstly, the ability of sera to inhibit the cytotoxic response does not correlate well with the peak

FIG. 3.—Velocity sedimentation profile of cytotoxic cells from MSV immune spleens at different times post MSV injection (shown above each panel in the figure). The fractions shown were suspended to the same volume and constant percentages of each fraction tested for their ability to lyse $^{51}$Cr-SDLC cells. All data points are means ($\pm$ standard error) of triplicate determinations using $5 \times 10^4$ target cells and 10% of the cells from each fraction. Data points to the left of each panel represent unfractionated spleen cells (tested at a 200 : 1 ratio).

growth of the tumour or the development of cells suppressing effector cells in other tests of CMI (Gorczynski, 1974*c*). However, development of serum blocking factors for this test of CMI parallels the development of serum blocking factors for other tests of CMI to MSV-TAA (Gorczynski *et al.*, 1975). Secondly, the actual blocking seen varies predictably according to the cytotoxicity seen with the unblocked cells (see upper panel of this figure). Finally, early progressor

FIG. 4.—Specificity of blocking to cytotoxicity (to SDLC or C57Bl spleen cells) by sera taken from mice at different times post MSV infection. Percent cytotoxicity to SDLC (closed circles) or C57Bl spleen cells (open circles) was determined in the presence or absence of sera (final concentration in test was 2%) taken from mice at various times post MSV infection (the number of mice used to prepare each serum pool is shown in brackets in the lower panel of the figure). The percent blocking of cytotoxicity was determined as:

$$100 \times \left(I - \frac{\text{Percent specific release in presence of serum}}{\text{Percent specific release in absence of serum}}\right)$$

The effector cells used in the test were from mice inoculated with $50 \times 10^6$ C57Bl spleen cells 20 days earlier followed by MSV 10 days later. The upper panel of the figure represents blocking with various effector cell numbers and a given serum (18 days post MSV). All sera in general showed similar effects, with the exception of early MSV sera (see figure) which often showed enhanced cytotoxicity. This is described in detail elsewhere (Gorczynski, in preparation). The lower panel shows the blocking phenomenon obtained with different sera and a constant effector : target ratio (250 : 1). In the absence of any sera the percent specific cytotoxicity to SDLC and C57Bl spleen cells at this effector : target ratio was $32 \pm 2 \cdot 2$ and $46 \pm 3 \cdot 1$ respectively.

serum (7–11 days post MSV) seems to enhance the cytotoxic response (see lower panel of Fig. 4). This effect, which may be a reflection of antibody mediated cellular cytotoxicity, will be discussed in a forthcoming communication.

Our earlier work (Gorczynski *et al.*, 1975) showed that the blocking activity seen with 20-day MSV serum was partially removed by preabsorption of the serum on an anti-MIG column (see Materials and Methods). The data of Table II repeat this finding as well as investigate the ability of various purified MSV antigens to block CMI from 11-day MSV spleen cells. The antigens used in this test were already shown to stimulate immune T lymphocytes from MSV regressor mice (Knight and Gorczynski, 1975). Spleen cells were pooled from 4 mice given MSV 11 days earlier and the percent specific lysis seen with these cells (and $^{51}$Cr-SDLC cells) was compared in the presence or absence of various concentrations of the antigens (and serum) under test. The data shown in Table II represent the blocking of cytotoxicity at a 100 : 1 effector : target ratio in

TABLE II.—*Serum and Viral Antigen Induced Blocking of Cytotoxicity to SDLC Cells*

| Source of blocking activity (concentration)* | | Percent specific $^{51}$Cr release† | Percent blocking of specific $^{51}$Cr release‡ |
|---|---|---|---|
| None | | 42±2·1 | — |
| VEA | 100 μg/ml | 41±1·8 | 2 |
| | 10 μg/ml | 42±1·9 | 0 |
| MSV(M) | 100 μg/ml | 42±1·1 | 0 |
| | 10 μg/m | 41±0·9 | 2 |
| MSV(G) | 100 μg/ml | 43±1·2 | −2 |
| | 10 μg/ml | 42±1·2 | 0 |
| Disrupted MSV(M) | 100 μg/ml | 33±1·4 | 21 |
| | 10 μg/ml | 39±1·3 | 7 |
| Disrupted MSV(G) | 150 μg/ml | 30±1·9 | 28 |
| | 50 μg/ml | 38±1·8 | 9 |
| Purified p30 protein | 140 μg/ml | 27±2·1 | 35 |
| | 40 μg/ml | 35±1·9 | 17 |
| C-Vgs protein | 200 μg/ml | 19±1·4 | 55 |
| | 50 μg/ml | 32±1·6 | 24 |
| 20-day MSV unabsorbed | 2% | 12±1·5 | 72 |
| serum | 0·4% | 27±2·1 | 36 |
| Anti-MIg column absorbed | | | |
| Column effluent | Equivalent volumes to unabsorbed material | 25±2·1<br>36±1·9 | 40<br>14 |
| Column absorbed-acid eluted | Equivalent volumes to unabsorbed material | 23±1·7<br>32±1·5 | 45<br>24 |
| Recombined fractions | Equivalent volumes to unabsorbed material | 15±1·5<br>29±1·9 | 64<br>31 |

* All cultures were set up containing 5 × $10^4$ $^{51}$Cr-SDLC cells and 5 × $10^6$ MSV spleen cells (from mice given MSV 11 days earlier). In addition, the incubation medium contained the various factors shown, whose blocking activity was under test, at two different concentrations (see Table II). The preparation of all these materials is described in detail in the text and elsewhere (Knight and Gorczynski, 1975). Twenty-day MSV serum was used before and after absorption on an anti-MIg column. In this case all column treated materials (*i.e.* effluent, absorbed and acid-eluted, and the recombined fractions) were concentrated by vacuum dialysis to their equivalent starting volume prior to test.

† Percent specific $^{51}$Cr release from SDLC cells after 15 h of incubation. All figures are arithmetic means (±1 standard error) of 3 cultures.

‡ Percent blocking of specific $^{51}$Cr release (in the absence of blocking factors) by the blocking factors shown in the first column.

a 15 h $^{51}Cr$ release assay with SDLC cells.

While cells responsive to whole MSV(M) and VEA exist at this time after sensitization (Knight and Gorczynski, 1974), it is clear from Table II that the cytotoxicity to $^{51}Cr$-labelled SDLC cells is not blocked by these purified viral antigens nor by the purified Gross virus pseudotype of MSV(M), MSV(G). However, MSV(M) and MSV(G), disrupted by the detergent Triton X-100, are roughly equipotent inhibitors of CMC, suggesting that cytotoxic lymphocytes recognize internal group specific viral antigens on the target cell surface. The major such antigen, p30, purified from MSV(M), inhibits CMC although we have not tested higher concentrations of this antigen to determine whether cytotoxicity could be totally inhibited. A much more potent inhibitor was extracted by papain digestion from SDLC cells themselves (C-Vgs). These data agree well with our earlier findings investigating the relative importance of sensitization to the respective MSV tumour associated antigens for *in vivo* tumour regression (Gorczynski and Knight, 1975). Moreover, we were able to repeat the findings of an earlier report that the blocking activity in 20-day MSV serum could be absorbed on (and eluted from) an anti-MIg column (Gorczynski *et al.*, 1975). With the data showing that blocking with MSV sera was enhanced when column effluent and acid eluted material were recombined (see Table II), we feel that blocking in these sera is probably mediated by antigen and/or antigen–antibody complexes.

TABLE III.—*Ability of C-Vgs and 20-day MSV Serum to Block Cytotoxicity from Effector Cells taken from Mice at Different Times post MSV*

| Source of effector cells* | Ratio to get 30% specific cytotoxicity† | Percent blocking by‡ C-Vgs (150 μg/ml) | Percent blocking by‡ 20-day MSV serum (2%) |
|---|---|---|---|
| 10 days post MSV | 85 : 1 | 62±2·3 | 88±3·2 |
| 16 days post MSV | 240 : 1 | 68±2·8 | 52±2·8 |
| 23 days post MSV | 150 : 1 | 60±3·2 | 51±2·7 |
| 30 days post MSV | 190 : 1 | 61±2·8 | 38±2·4 |

* Spleen cells were pooled from 4 mice within each group given MSV at the times shown. The cells were then tested at various doses for their cytotoxicity to $5 \times 10^4$ $^{51}Cr$-SDLC cells, in the presence or absence of the blocking factors shown in the final two columns.

† Ratio of effector cells (shown in the first column) to target cells ($^{51}Cr$-SDLC cells) to get 30% specific lysis at 15 h.

‡ Percent blocking of the cytotoxicity caused by that ratio of cells shown in the 2nd column by either C-Vgs (at 150 μg/ml) or 20-day post MSV serum (at 2% final concentration). All data points were determined from triplicate cultures in the presence or absence of blocking factors and the standard errors shown were computed from the variation in both numerator and denominator.

*Blocking of different populations of effector cells by 20-day MSV sera*

Having described here (Fig. 3) and elsewhere (Knight and Gorczynski, 1974) the heterogeneity of effector cells to MSV-TAA, we were interested in the ability of these different populations to be blocked by MSV blocking sera. Accordingly we performed two types of experiment.

In the first we took cells from animals of different times post MSV infection and investigated the cytotoxicity of these cells at various effector cell : target cell ratios in the presence or absence of a standard concentration of 20-day MSV serum (2%) or C-Vgs (150 μg/ml). The blocking seen with that ratio of cells giving the same percent specific cytotoxicity in the absence of serum (a function of different effector : target cell ratios for the different populations) was then determined. These data, together with the respective ratios giving the same cytotoxicity (30%) in the unblocked population, are indicated in Table III. Whereas C-Vgs apparently blocks effector cells from mice at all times post MSV infection, 20-day MSV serum was less able to block the cytotoxicity of effector cells later in the response.

In a second experiment, we fractionated cells by velocity sedimentation from animals given MSV 12 days earlier and tested the fractions at different ratios in the presence or absence of a standard concentration of 20-day MSV serum (2%) or C-Vgs (150 $\mu$g/ml).

$10 \times 10^8$ spleen cells from mice given MSV 11 days earlier were sedimented for 4 h at 4°C and fractions sedimenting with the velocities shown in Fig. 5 were pooled and centrifuged as before. The recovered cells were resuspended in $DF_{10}$, recounted, and cultured with $5 \times 10^4$ $^{51}$Cr-SDLC cells at various effector : target ratios, in the presence and absence of C-Vgs (150 $\mu$g/ml) or 20-day MSV serum (2%). After 15 h the supernatants were harvested and the percent specific $^{51}$Cr release from the various fractions and an unfractionated control sample were calculated as before. The percent blocking of this activity was calculated from that ratio of cells which, from all fractions, gave 30% specific release in the absence of blocking factors. Once again this is a valid comparison provided (as here) the titration curves from the respective fractions are parallel.

The data of Fig. 5 show a good correlation between the ability of both C-Vgs and 20-day MSV serum to block the cytotoxicity from different subpopulations of lymphocytes. Nevertheless, the most interesting feature is the demonstration that a population of cytotoxic T lymphocytes (see Table I) exists which is not appreciably blocked by either serum or C-Vgs. As in Table III, the inhibition by serum, where demonstrable, was generally less than that mediated by C-Vgs. Since we have shown that at least some of the serum mediated inhibition is due to factors which bind to an anti-immunoglobulin column (Gorczynski *et al.*, 1975), this suggests that antibody (perhaps complexes with antigen) is a less potent blocking reagent than free TAA. Alternatively, the differences may be caused by different concentrations of blocking materials in these preparations. In view of the existence of a non-blockable fraction of cytotoxic T lymphocytes in the spleens of MSV progressor animals (see Fig. 5) and the decreased ability of cytotoxic spleen cells of MSV regressor animals to be blocked (Table III), it is tempting to speculate that non-blockable T cells are in some way responsible for tumour regression.

As a final test of the physiological relevance of this *in vitro* demonstration of blocking of cytotoxic lymphocytes, we investigated the effect of overnight incubation in $DF_{10}$ followed by similar incubation in blocking sera on the cytotoxic potential of spleen cells taken from mice at different times post MSV inoculation. The rationale behind this approach was as follows: (1) The kinetics of development of cytotoxic cells (see Fig. 1) suggested that some *in vivo* blocking of effector cells may be taking place late in tumour progression (days 15–18); (2) earlier reports (de Landazuri and Herberman, 1972; Gorczynski and Tigelaar, 1975) had suggested that *in vivo* blocked cells in different tumour systems become activated for cytotoxicity after overnight incubation.

Spleen cells were pooled aseptically from groups of 5 mice inoculated with MSV at different times and aliquots of each pool were tested at different ratios for their cytotoxicity with $5 \times 10^4$ $^{51}$Cr-SDLC target cells. The remainder ($6 \times 10^8$) of the cells were incubated for 15 h in $DF_{10}$ in small Marbrook type culture vessels at a concentration of $2 \times 10^7$ cells per ml. After this time the cultures were harvested, the cells washed twice in $DF_{10}$ and aliquots retested for cytotoxicity with $5 \times 10^4$ $^{51}$Cr-SDLC target cells, before and after treatment with anti-Br-$\theta$ (see Materials and Methods). This latter test was performed to check that the cytotoxicity after overnight incubation in $DF_{10}$ was still due wholly to T lymphocytes. The remainder of the harvested cells ($1{\cdot}5 \times 10^8$) were divided into 3 pools and $50 \times 10^6$ cells of each incubated for a further 15 h

FIG. 5.—Blocking of cytotoxicity from velocity sedimented 11-day MSV immune spleen cells by C-Vgs (150 μg/ml—upper panel) or 20-day post MSV serum (2% final concentration—lower panel). The computation of these data are explained in detail in the text. All points represent arithmetic means of 3 cultures (in the presence or absence of blocking factors). The standard error bars were computed taking into account the variation in both the numerator and denominator. Data to the left of each panel show the blocking of unfractionated cells.

in either $DF_{10}$ or $DF_{10}$ containing 10% normal Balb/c serum or 10% 20-day post MSV Balb/c serum. After this time the cells were harvested, washed twice in $DF_{10}$ as before and their cytotoxic potential with $5 \times 10^4$ $^{51}$Cr-SDLC target cells measured once more. The data from these three time points of assay are shown in Table IV. At each time of harvesting the mean recovery of viable cells (after 15 h) was 55 ± 10%. The data are expressed as the ratio of

TABLE IV.—*Unblocking and Re-blocking of* in vivo *Blocked MSV Immune Spleen Cells*

| Source of effector cells* | Specific cytotoxic potential† (Day 0) | Specific cytotoxic potential after incubation in $DF_{10}$‡ | | Specific cytotoxic potential after incubation in§ | | |
|---|---|---|---|---|---|---|
| | | Untreated | Anti-Br-θ treated | $DF_{10}$ | 10% normal serum | 10% 20-day MSV serum |
| 10 days post MSV | 110 : 1 | 70 : 1 | $0\cdot9\pm0\cdot5$ | 40 : 1 | 45 : 1 | 380 : 1 |
| 16 days post MSV | 340 : 1 | 50 : 1 | $-1\cdot6\pm0\cdot9$ | 30 : 1 | 40 : 1 | 300 : 1 |
| 23 days post MSV | 210 : 1 | 70 : 1 | $0\cdot4\pm1\cdot8$ | 50 : 1 | 60 : 1 | 270 : 1 |
| 30 days post MSV | | | | | | |
| 30 days post MSV | 290 : 1 | 80 : 1 | $1\cdot1\pm2\cdot2$ | 60 : 1 | 70 : 1 | 250 : 1 |

* Effector cells were pooled from groups of 5 mice injected with MSV at the times shown. The cells were tested at different ratios for their cytotoxicity to $5 \times 10^4$ $^{51}$Cr-labelled SDLC cells.

† Ratio of effector : target cells needed to see 25% specific $^{51}$Cr release from $^{51}$Cr-SDLC cells, using spleen cells freshly removed from mice (see first column).

‡ Ratio of effector : target cells needed to see 25% specific $^{51}$Cr release from $^{51}$Cr-SDLC cells, using spleen cells which had been incubated in $DF_{10}$ for 15 h. The cytotoxicity shown for anti-Br-θ + complement treated incubated cells is for the same ratio of untreated cells giving 25% specific release.

§ Ratio of effector : target cells needed to see 25% specific $^{51}$Cr release from $^{51}$Cr-SDLC cells, using spleen cells which were first incubated for 15 h in $DF^{ac}$ (see third column) and then were incubated for a further 15 h in either $DF_{10}$ or $DF_{10}$ containing 10% normal serum or 10% 20-day post MSV serum.

effector : target cells needed to produce 25% specific $^{51}$Cr release with each pool of cells, except for the anti-Br-θ treated cells, where the cytotoxicity seen at that ratio of untreated cells giving 25% specific release is shown.

Comparison of the columns of Table IV reveals some interesting features. Cytotoxic activities from all pools of cells tested were increased by incubation in $DF_{10}$, as indicated by the lower effector : target cell ratio needed to produce the same degree of lysis. After the incubation, the cytotoxicity remained wholly due to cytotoxic T lymphocytes. The possibility that small increases in cytotoxic potential might be explained by selective survival of cytotoxic cells (since viable cell recovery was only 55 ± 10%) must be borne in mind (see also Gorczynski and Tigelaar, 1975). The very dramatic increase in activity seen with 16-, 23- and 30-day post MSV-spleen cells, which now become as active as 10-day post MSV cells treated similarly, is not, we feel, explainable by selective survival. Rather, this is very suggestive of the "leaching off" of blocking materials from the cell surface during the incubation.

The effect of further incubation in either $DF_{10}$, normal mouse serum or blocking serum (see Fig. 4 and 5 and Tables II and III) was even more striking. After incubation in 20-day MSV serum the cytotoxic potential of the cells was decreased to similar levels to (or even less than) the initial values obtained directly after removing the spleen cells from the intact animals. These data very strongly argue that the *in vivo* blocking effect is mediated by serum factors. Moreover, since such blocking was produced in the absence of target cells, the data suggest that the blocking factors are either free antigen or antigen–antibody complexes, each of which would be capable of binding to cytotoxic lymphocyte receptors.

### DISCUSSION

The main emphasis of this work has been to analyse the ability of MSV tumour associated antigens (TAA) to block the direct cell mediated cytotoxicity significance of this phenomenon. Our initial studies suggested that the effector cells in the cytotoxic assay we employed (a 15 h $^{51}$Cr release assay) were antigen specific cytotoxic T cells. This was so irrespective of the time post MSV infection at which spleen cells (containing effector cells) were harvested from immune mice (Table I and Fig. 2). These results concur with the studies of Herberman *et al.* (1973) who studied immunity to MSV in C57Bl mice, and with those of Leclerc *et al.* (1973), both of whom used a similar $^{51}$Cr release assay. They do not agree, however, with the results of Lamon *et al.* (1972, 1973) who found in the MCA evidence for a non-T cytotoxic cell at late times post MSV. However, these discrepancies may be related to the type of assay used for studying CMC, the surface antigens expressed by the target cells used and the length of the assay.

As defined by velocity sedimentation (Fig. 3), tumour cell specificity (Fig. 2) and the effects of an anti-T (rabbit anti-Br-$\theta$) or anti-B (rabbit anti-mouse immunoglobulin) antiserum (Table I) the effector cells in the $^{51}$Cr release assay correspond well with those defined in other tests of CMI in the MSV system (Gorczynski, 1974*a*). Moreover, when purified virally induced TAA were tested for their ability to inhibit cytotoxicity, the results substantially confirmed earlier data on blocking in other tests of CMI (Gorczynski *et al.*, 1974*b*) and the relative importance of defined virally induced TAA in *in vivo* tumour rejection (see Table II and Knight and Gorczynski, 1975). For example, the cell extract, C-Vgs, was both the most potent inhibitor of CMC and the most effective antigen for sensitizing cells capable, on adoptive transfer, of protecting sublethally irradiated mice against MSV tumour challenge (Gorczynski and Knight, 1975). Similarly, the viral group specific antigen, p30, inhibited CMC, stimulated blast transformation by regressor T lymphocytes (Knight and Gorczynski, 1975) and sensitized cells which, on transfer, protected against MSV tumour challenge *in vivo*. Cell extracts prepared from tumour induced by several strains of MLV have also been shown to inhibit MSV-M immune CMC in a MCA (Plata and Levy, 1974).

The data of Fig. 4, using spleen cells from mice sensitized to both MSV-TAA and to murine alloantigens, indicate that the blocking mediated by soluble TAA (C-Vgs) or serum (20-day post MSV serum—see Table II) was antigen specific. In Table II and elsewhere (Gorczynski *et al.*, 1975) we found that the serum mediated inhibition was in part due to material not absorbed on an anti-MIg column (free TAA?) and in part to material absorbed to the column. Far greater blocking was seen when these two components were mixed, however, suggesting that the most potent blockade was mediated by antigen–antibody complexes in antigen excess.

When comparing these data with the *in vivo* growth pattern of the tumour, several anomalies become apparent. Firstly, cytotoxicity measured by the CRT by spleen cells from tumour bearing mice (Days 11–15) was greater than from late progressors or early regressors (see Fig. 1). This correlates well with the development of serum blocking factors (Fig. 4) but not with tumour growth. Secondly, in earlier studies (Gorczynski, 1974*c*) we described how the CMI (measured by lymphocyte transformation or macrophage migration inhibition) decreased during the growth phrase of the tumour, a decrease associated with the development of a suppressor cell population (see also Kirchner *et al.*, 1974; Kilburn *et al.*, 1974). Yet this was not the case with CMI measured by $^{51}$Cr release (Fig. 1), nor were factors (antigen–antibody complexes?) released by these suppressor cells which could block CMI in a $^{51}$Cr assay, though such factors *were* released to block other assays of CMI (Gorczynski *et al.*, 1975).

To clarify the relationship between *in vivo* tumour growth and *in vitro* CMI, we tested the effect of antigen and 20-day immune serum on fractions of spleen cells prepared by velocity sedimentation, from mice injected with MSV at one specific time and on unfractionated spleen cells from mice injected with MSV at different times. We also investigated the effect of incubating immune cells in medium before assay and the effect of antigen and 20-day immune serum on these *in vitro* activated cells.

The data from these experiments are detailed in Tables III and IV and Fig. 5. In brief, we found that all unfractionated spleen cells tested showed a similar response (in terms of facility to be blocked) to soluble MSV-TAA (C-Vgs), but a widely different response to serum blocking factors. In particular, spleen cells showed a marked decline in their ability to be blocked at around the time of tumour regression, and the decrease was maintained in regressor animals (Table III). When spleen cells from MSV progressor animals were fractionated, a population of cells was identified which was refractory to blocking by C-Vgs and serum. It is interesting to note that this pool of cytotoxic non-blockable T cells co-sediments with the suppressor cells earlier described (Gorczynski, 1974*c*). This may help to explain why suppressor cells and their soluble products had no effect on CMI analysed by a $^{51}$Cr test. Finally, as described by de Landazuri and Herberman (1972) for a rat lymphoma, we found that Balb/c MSV regressor animals contained spleen cells whose cytotoxic potential was markedly increased by overnight incubation in medium. However, and equally interesting, the same was also true of MSV late progressor and early regressor animals (Table IV). Again, as determined by treatment with anti-Br-$\theta$, the cytotoxic cells after overnight incubation were T lymphocytes (see Table IV). The increase in cytotoxic activity after incubation *in vitro* could be abolished if the cells were now reincubated in blocking serum (Table IV).

Although, therefore, the cytotoxicity of unfractionated immune spleen cells taken between 10 and 30 days after MSV injection can be inhibited by antigen and by 20-day serum, a subpopulation can be fractionated by velocity sedimentation which is cytotoxic, but which is refractory to inhibition by these reagents. These non-inhibitable lymphocytes may indeed be the subpopulation most relevant to the tumour status of the animal, particularly from the time when the animal's serum becomes inhibitory to other cytotoxic cells. Since this cell population co-sediments with non-T cells which suppress other parameters of CMI (Gorczynski, 1974*c*), however, the suppressor cell product, rather than inhibiting direct cytotoxicity, may bind to the cytotoxic T cell surface so as to prevent the binding of antigen or blocking serum. There is evidence, however, that suppressor cells can reduce the protective effect of MSV regressor T lymphocytes adoptively transferred to sublethally irradiated mice (Gorczynski and Norbury, 1974).

We gratefully acknowledge the expert technical assistance of K. Adams and C. Norbury.

### REFERENCES

AOKI, T., HERBERMAN, R. B., JOHNSON, P. A., LILL, M. & STURM, M. M. (1972) Wild-type Growth Leukaemia Virus: Classification of Soluble Antigens and (GSA). *J. Virol.*, **10**, 1208.

BOYLE, W. (1968) An Extension of the $^{51}$Cr-release Assay for the Estimation of Mouse Cytotoxins. *Transplantation*, **6**, 761.

CEROTTINI, J. C., NORDIN, A. A. & BRUNNER, K. T. (1970) Specific *in vitro* Cytotoxicity of Thymus Derived Sensitized to Lymphocytes Alloantigens. *Nature, Lond.*, **228**, 1308.

ECKNER, R. J. & STEEVES, R. A. (1972) A Classification of the Murine Leukemia Viruses. Neutralization of Pseudotypes of Friend Spleen Focus-forming Virus by Type Specific Murine Antisera. *J. exp. Med.*, **136**, 832.

FEFER, A., MCCOY, J. L., PARK, K. & GLYNN, J. P. (1968) Immunologic, Virologic and Pathologic Studies of Regression of Authochthonous Moloney Sarcoma Virus induced Tumors in Mice. *Cancer Res.*, **28**, 1577.

GELDERBLOM, H., BAUER, H. & GRAF, T. (1972) Cell-surface Antigens Induced by Avian RNA Tumour Viruses: Detection by Immunoferritin Technique. *Virology*, **47**, 416.

GORCZYNSKI, R. M. (1974*a*) Immunity to Murine Sarcoma Virus-induced Tumors. 1. Specific T Lymphocytes active in Macrophage Migration Inhibition and Lymphocyte Transformation. *J. Immun.*, **112**, 1815.

GORCZYNSKI, R. M. (1974*b*) Evidence for *in vivo* Protection against Murine Sarcoma Virus induced Tumors by T lymphocytes from Immune Animals. *J. Immun.*, **112**, 533.

GORCZYNSKI, R. M. (1974*c*) Immunity to Murine Sarcoma Virus-induced Tumors. II. Suppression of T Cell-mediated Immunity by Cells from Progressor Animals. *J. Immun.*, **112**, 1826.

GORCZYNSKI, R. M., KONTAINEN, S., MITCHISON, N. A. & TIGELAAR, R. E. (1974) Antigen-antibody Complexes as Blocking Factors on the T Lymphocyte Surface. In *Cellular Selection and Regulation in the Immune Response*. Ed. G. M. Edelman. New York: Raven Press. p. 143.

GORCZYNSKI, R. M., KILBURN, D. G., KNIGHT, R. A., NORBURY, C., PARKER, D. C. & SMITH, J. B. (1975) Non-specific and Specific Immunosuppression in Tumour Bearing Mice by Soluble Immune Complexes. *Nature, Lond.* In the press.

GORCZYNSKI, R. M. & KNIGHT, R. A. (1975) Cell-mediated Immunity to Moloney Sarcoma Virus in Mice. II. Analysis of Antigenic Specificities Involved in T Lymphocyte Mediated *in vivo* Rejection of Murine Sarcoma Virus (MSV)-induced Tumours. *Eur. J. Immunol.* In the press.

GORCZYNSKI, R. M. & NORBURY, C. (1974) Immunity to Murine Sarcoma Virus induced Tumours. III. Analysis of the Cell Populations Involved in Protection from Lethal Tumour Progression of Sublethally Irradiated MSV Inoculated Mice. *Br. J. Cancer*, **30**, 118.

GORCZYNSKI, R. M. & TIGELAAR, R. E. (1975) Unmasking of T Cell Mediated Immunity to Tumour Allografts. *Cell. Immunol.* In the press.

HELLSTRÖM, I. & HELLSTRÖM, K. E. (1969) Studies on Cell-mediated Immunity and its Serum-mediated Inhibition in Moloney-virus induced Mouse Sarcoma. *Int. J. Cancer*, **5**, 587.

HERBERMAN, R. B., NUNN, M. E., LABRIN, D. H. & ASOFSKY, R. (1973) Effector of Antibody to $\theta$ Antigen on Cell-mediated Immunity in Syngeneic Mice by Murine Sarcoma Virus. *J. natn. Cancer Inst.*, **51**, 1509.

KILBURN, D. G., SMITH, J. B. & GORCZYNSKI, R. M. (1974) Nonspecific Suppression of T Lymphocyte Responses in Mice Carrying Progressively Growing Tumours. *Eur. J. Immunol.* **4**, 784.

KIRCHNER, H., CHUSED, T. M., HERBERMAN, R. B., HOLDEN, H. T. & LABRIN, D. H. (1974) Evidence of Suppressor Cell Activity in Spleens of Mice Bearing Primary Tumors induced by Molony Sarcoma Virus. *J. exp. Med.*, **139**, 1473.

KNIGHT, R. A. & GORCZYNSKI, R. M. (1975) Cell Mediated Immunity to Moloney Sarcoma Virus in Mice. I. Analysis of Antigens Responsible for Lymphocyte Stimulation in Regressor Mice. *Int. J. Cancer.* In the press.

KNIGHT, R. A., LEECH, S., MITCHISON, N. A. & SHELLMAN, G. (1974) Three Ways of using Immunology to Investigate the Cell Surface: Cocapping, Helper Determinants, and Immunogenicity. In VIIth Miles Symposium, Vienna, Johns Hopkins University Press. p. 425.

KURTH, R. & BAUER, H. (1972) Cell-surface Antigens Induced by Avian RNA Tumour Viruses: Detection by a Cytotoxic Micro-assay. *Virology*, **47**, 426.

LAMON, E. W., SKURZAK, H. M., KLEIN, E. & WIGZELL, H. (1972) *In vitro* Cytotoxicity by a Non Thymus-processed Lymphocyte Population with Specificity for a Virally Determined Tumour Cell Surface Antigen. *J. exp. Med.*, **136**, 1072.

LAMON, E. W., WIGZELL, H., KLEIN, E., ANDERSSON, B. & SKURZAK, H. M. (1973) The Lymphocyte Response to Primary Moloney Sarcoma Virus Tumours in Balb/c Mice. Definition of the Active Sub-populations at Different Times after Infection. *J. exp. Med.*, **137**, 1472.

DE LANDAZURI, M. O. & HERBERMANN, R. B (1972) Immune Response to Gross Virus Induced Lymphoma. III. Characteristics of the Cellular Immune Response. *J. natn. Cancer Inst.*, **49**, 147.

LAW, L. W. & APPELA, E. (1973) Immunogenic Properties of Solubilized Tumour Antigen from an RNA Virus Transformed Neoplasm. *Nature, Lond.*, **243**, 83.

LECLERC, J. C., GOMARD, E., PLATA, F. & LEVY, J. R. (1973) Cell-mediated Immune Reaction against Tumours by Oncornaviruses. II. Nature of the Effector Cells in Tumour Cell Cytolysis. *Int. J. Cancer*, **11**, 426.

LOWRY, O. H., ROSEBROUGH, N. J., FARR, A. L. & RANDALL, R. J. (1951) Protein Measurement with the Folin Phenol Reagent. *J. biol. Chem.*, **193**, 265.

MACLENNAN, I. C. M., LOEWI, G. & HARDING, B. (1970) The Role of Immunoglobulins in Lymphocyte Mediated Cell Damage *in vitro*. 1. Comparison of the Effects of Target Cell Specific Antibody and Normal Serum Factors on Cellular Damage by Immune and Non-immune Lymphocytes. *Immunology*, **18**, 397.

MILLER, R. G. & PHILLIPS, R. A. (1969) Separation of Cells by Velocity Sedimentation. *J. cell. Physiol.*, **73**, 191.

PLATA, F. & LEVY, J. P. (1974) Comparative Studies on the Blocking of Syngeneic Effector T Cells by Soluble Tumour Antigens. *Nature, Lond.*, **249**, 271.

SHELLAM, G. & KNIGHT, R. A. (1974) Inhibition of Cell Mediated Cytotoxicity against Tumour Cells by Antigen or Serum from Tumour Bearing Rats. *Nature, Lond.* **252**, 330.

SJÖGREN, H. O., HELLSTRÖM, I., BANSAL, S. C. & HELLSTRÖM, K. E. (1971) Suggestive Evidence that the Blocking Antibodies of Tumor-bearing Individuals may be Antigen–antibody Complexes. *Proc. natn. Acad. Sci. U.S.A.*, **68**, 1372.

YOSHIKI, T., MELLORS, R. C., HARDY, W. D. & FLEISSNER, E. (1974) Common Cell Surface Antigen associated with Mammalian C-type RNA Viruses. Cell Membrane-bound gs Antigen. *J. exp. Med.*, **139**, 925.